# Proposed Biosynthesis and Molecular Targets of Manzamine Alkaloids from *Acanthostrongylophora ingens* (Phylum Porifera: Class Demospongiae)

**DOI:** 10.3390/md24090313

**Published:** 2026-09-07

**Authors:** Novriyandi Hanif, Ritha Lusian Karuwal, Muhammad Naufal Khotmi Ramadhan Ashari Jaya Sudrajat, Didik Huswo Utomo, Gibral Abdul Khalik, Heri Yanti, Lik Tong Tan, Nicole J. de Voogd, Anggia Murni, Junichi Tanaka

**Affiliations:** 1 Department of Chemistry, Faculty of Mathematics and Natural Sciences, IPB University, Bogor 16680, Indonesia; ritha.karuwal@lecturer.unpatti.ac.id (R.L.K.); naufalkrajsudrajat@apps.ipb.ac.id (M.N.K.R.A.J.S.); gibralabdul98@gmail.com (G.A.K.); heriyanti2103@gmail.com (H.Y.); 2 Tropical Biopharmaca Research Center, IPB University, Bogor 16128, Indonesia; anggia_murni@apps.ipb.ac.id; 3 Biology Education Program, Faculty of Teaching and Education, University of Pattimura, Ambon 97233, Indonesia; 4 Bioinformatics Research Center, Indonesian Institute of Bioinformatics (INBIO Indonesia), Malang 65145, Indonesia; didik.huswo@gmail.com; 5 Natural Sciences and Science Education, National Institute of Education, Nanyang Technological University, 1 Nanyang Walk, Singapore 637616, Singapore; liktong.tan@nie.edu.sg; 6 Institute of Biology Leiden (IBL), Leiden University, 2300 RA Leiden, The Netherlands; nicole.devoogd@naturalis.nl; 7 Naturalis Biodiversity Center, 2333 BE Leiden, The Netherlands; 8 Department of Chemistry, Biology and Marine Science, University of the Ryukyus, Okinawa 903-0213, Japan; jtanaka@cs.u-ryukyu.ac.jp

**Keywords:** bioinformatics, biosynthesis, molecular networking, molecular target, NMR calculation

## Abstract

Manzamine alkaloids are a structurally diverse class of marine-derived alkaloids that have attracted considerable interest because of their complex polycyclic architectures, diverse biological activities, and distinctive biosynthetic features. The Indonesian marine sponge *Acanthostrongylophora ingens* is a particularly rich source of these alkaloids, providing an opportunity to further explore their chemical diversity, biosynthetic relationships, and biological properties. Detailed analyses of LC-HR-ESI-MS/MS and molecular networking were performed on EtOAc layers obtained from H_2_O/EtOAc partitioning of methanolic extracts of the marine sponge, *Acanthostrongylophora ingens*, collected from Raja Ampat (Southwest Papua), the Thousand Islands reef complex (Jakarta Special Region), Spermonde Archipelago (South Sulawesi), and Sangiang Island (Banten). This approach revealed the presence of both known and new manzamine alkaloids, along with their possible precursors and structural modifications, thereby supporting the proposed biosynthetic origin of these alkaloids. In addition, three flexible cytotoxic manzamine alkaloids, namely (+)-manzamine A hydrochloride (**1**), (+)-manzamine B (**2**), and (+)-32,33-dihydro-31-hydroxymanzamine A (**3**), were purified and their relative configurations determined using quantum mechanic NMR-based calculations and validated by X-ray analysis. (+)-Manzamine A hydrochloride (**1**) displayed the most potent cytotoxic activities against *Artemia salina* larvae (LC_50_ value of 0.041 ± 0.012 µM) and HEK293T cells (IC_50_ value of 0.599 ± 0.057 µM). The potent cytotoxic activity suggested the importance of azocine ring (*Z*-olefin at C32–C33) in **1**. Molecular mechanisms of **1**–**3** were subsequently revealed through computational approaches identifying GSK-3β as a potential target. Docking results indicated that **3** possessed better binding affinity to GSK-3β, compared with other ligands. All isolated manzamines (**1**–**3**) were found to be stable in the binding site. A molecular mechanism is also proposed to provide insights into how manzamines interrupt cancer cells by targeting specifically with GSK-3β.

## 1. Introduction

**Figure 1:**
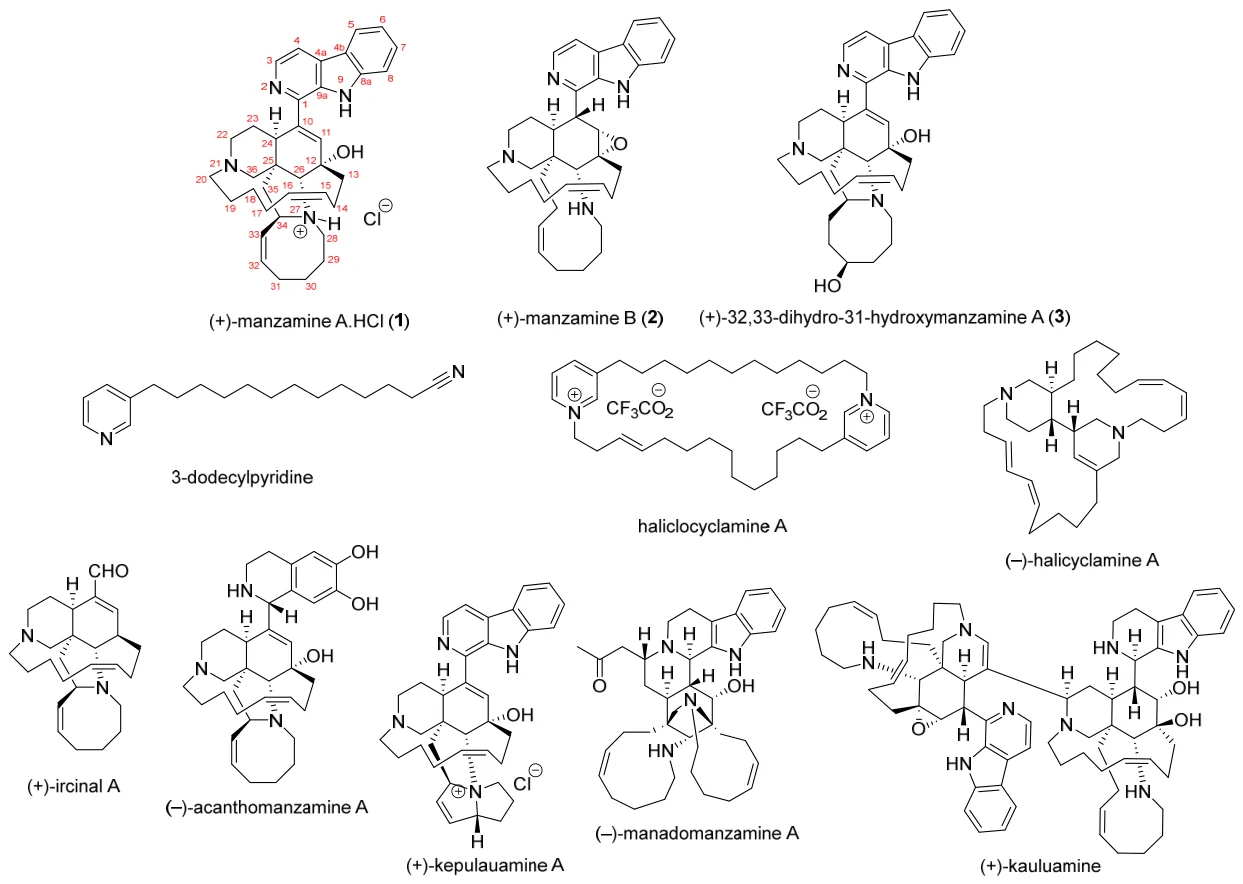
Representative manzamine alkaloids including possible biosynthetic precursors and manzamine product modifications.

The marine sponge *Acanthostrongylophora ingens*, a member of the family Petrosiidae, is a prolific source of bioactive manzamine alkaloids. These compounds are characterized by a complex polycyclic framework containing a β-carboline moiety and a unique 6/6/8/13-membered ring system, as represented by the first member of this family, manzamine A (**1**) [1]. To date, more than 200 members with varied structures of manzamine A (**1**), including its apparent precursors and its modifications, have been discovered, such as 3-alkyl pyridine represented by 3-dodecyl pyridine and haliclocyclamine A; halicyclamine exampled by (−)-halicyclamine A; and ircinal illustrated by (+)-ircinal A. Various structural modifications of (+)-manzamine A (**1**) could be seen in: β-carboline system, e.g., (−)-acanthomanzamine A; polycyclic ring system, e.g., (+)-manzamine B (**2**), (+)-32,33-dihydro-31-hydroxymanzamine A (**3**), (+)-kepulauamine A; mixed modification of β-carboline and polycyclic systems, e.g., (−)-manadomanzamine A; and dimerization, e.g., (+)-kauluamine (Figure 1) [2,3]. Although the true producer of manzamine A (**1**) from *A. ingens* was disclosed as a sponge-associated bacterium, *Micromonospora* sp., no manzamine biosynthetic gene clusters have been identified and no enzymes capable of transforming putative manzamine intermediates have been disclosed [4]. Therefore, structural analysis of manzamine A (**1**) and its related products is required to identify their biosynthetic origins and possible reactions involved. This could be disclosed by LC-HR-ESI-MS/MS and molecular networking analyses [5] from the EtOAc extracts of *A. ingens*. Moreover, the analyses allowed us to identify putative new molecules for future natural product discovery.

The manzamine alkaloids possess unique pharmacological activities, such as anticancer, antimicrobial, and antimalarial properties [6]. Karan and their co-workers highlighted manzamine A (**1**) as a promising marine-derived cancer therapeutic agent. Manzamine A (**1**) exhibits cytotoxic activity through multiple mechanisms, including regulation of the cell cycle, inhibition of cell migration, epithelial-to-mesenchymal transition (EMT), and induction of autophagy and apoptosis. These effects are mediated through interactions with several molecular targets and signaling pathways, including E2F transcriptional factors, ribosomal S6 kinases, androgen receptor (AR), SIX1, GSK-3β, v-ATPase, and p53/p21/p27 cascades [7]. Computational analysis revealed that manzamine A (**1**) preferably binds to the *N*-terminal kinase domain (NTKD) of RSK1 over the C-terminal domain (CTKD). The predicted binding energies for the NTKD and CTKD complexes with manzamine A (**1**) were −62.132 and −55.497 kcal/mol, respectively [8]. This result supported inhibitory effect of manzamine A to the 90 kDa ribosomal S6 kinase (RSK)1/2, particularly RSK1, in human cervical carcinoma cells, with IC_50_ values of 15.01 μM for RSK1 and 108.4 μM for RSK2 [8].

Another enzyme that plays a key role in regulating cell development, particularly cell cycle arrest and apoptosis, is GSK-3β [9]. In vitro studies have demonstrated the ability of manzamine A (**1**) to inhibit GSK-3β, leading to the induction of apoptosis in pancreatic cancer cells [10] and inhibitory effect in glioma (U87 and U373) cells [11]. Meanwhile, Hamann and their co-workers reported that manzamine A (**1**) inhibited human GSK-3β activity (73.2% at 25 µM). Moreover, manzamine A (**1**) was reported to inhibit GSK-3β-dependent tau protein phosphorylation on the basis of a cell-based assay [12,13]. To date, evidence of GSK-3β inhibition by manzamine A (**1**) is confined to in vitro studies, leaving its broader multi-target mechanism largely unexplored in vitro and in vivo. Yet, the compound’s proven ability to reduce cancer cell proliferation suggests it holds great potential for innovative drug combinations. Utilizing in silico approaches will be an essential next step to validate these multi-target interactions and guide future development. In silico studies on the binding of manzamine to GSK-3β remain limited. An example was shown by the docking study of manzamine A (**1**) to GSK-3β in lung cancer possessing an affinity of −10.7 kcal/mol [14]. This suggests a high binding strength and positions it as a potential target for further research.

All observed biological activities are driven by chemical structures, including molecular shape, symmetry and stereogenicity/chirality. Stereochemical determination of natural products could be significantly more challenging than those of synthetic organic compounds [15]. Three emerging strategies are currently developed to solve the planar and stereochemical problem of flexible molecules, including quantum computational chemistry, crystalline sponges, and biosynthetic gene-dependent discovery [16]. NMR calculation as one of the emerging strategies in quantum computational chemistry is an important approach for structure elucidation of natural products. The calculation models for determining NMR parameters, especially ^13^C NMR, have been used to assign relative configuration and corrections of reported structures that have been misassigned. An efficient method for calculating the ^13^C NMR chemical shifts in flexible natural products [17] has been applied with a variety of planar and stereostructures. Such strategy has been effectively used to determine the relative configuration of secondary alcohol attached at 7- and 11-membering ring in antheliol and sangiangol B, respectively [18].

In our quest for bioactive compounds from Indonesian marine organisms [19], we have analyzed the organic layers prepared from H_2_O/EtOAc partitioning of extracts of *A. ingens* samples collected from various locations. In particular, preliminary NMR analysis indicated that the organic layers were enriched in manzamine alkaloids, which correlated with their potent cytotoxicity against *A. salina* larvae, with an LC_50_ value of 0.16 µg/mL. This initial result motivated us to investigate the plausible biosynthetic pathway and presence of modified products of manzamine alkaloids through analyses of LC-HR-ESI-MS/MS and molecular networking of four *A. ingens* specimens collected from Raja Ampat (Southwest Papua), the Thousand Islands (Jakarta Special Region), Spermonde Archipelago (South Sulawesi), and Sangiang Island (Banten). As an additional means of validating the stereochemical assignments previously established through spectroscopic and other experimental studies, quantum mechanical NMR-based calculations were employed in the present study to independently evaluate the consistency of the proposed configurations with the observed NMR data and structural features. Moreover, structure–activity relationship (SAR) of these molecules was established using *A. salina*, human immortal kidney cells (HEK293T), Gram-positive bacteria *Staphylococcus aureus*, Gram-negative bacteria *Escherichia coli*, and α-glucosidase enzyme. Furthermore, molecular mechanisms of **1**–**3** were investigated through computational approaches to rationalize the observed activity results and suggest new molecular targets of **1**.

## 2. Result and Discussion

To clarify the nature of the samples analyzed in this study, the LC-HR-ESI-MS/MS and molecular networking analyses were performed on the EtOAc layers obtained from solvent partitioning of extracts of *Acanthostrongylophora ingens* samples collected from four different locations in Indonesia. This partitioning strategy performed on the extracts was intended to enrich the lipophilic and moderately polar secondary metabolites, including the manzamine alkaloids, prior to mass spectrometric analysis. For the isolation and structural characterization of individual manzamine alkaloids, separate, larger-scale *A. ingens* specimens were subjected to ethanolic extraction followed by solvent partitioning and chromatographic separation. This led to the isolation of (+)-manzamine A hydrochloride (**1**), (+)-manzamine B (**2**), and (+)-32,33-dihydro-31-hydroxymanzamine A (**3**).

**Figure 2:**
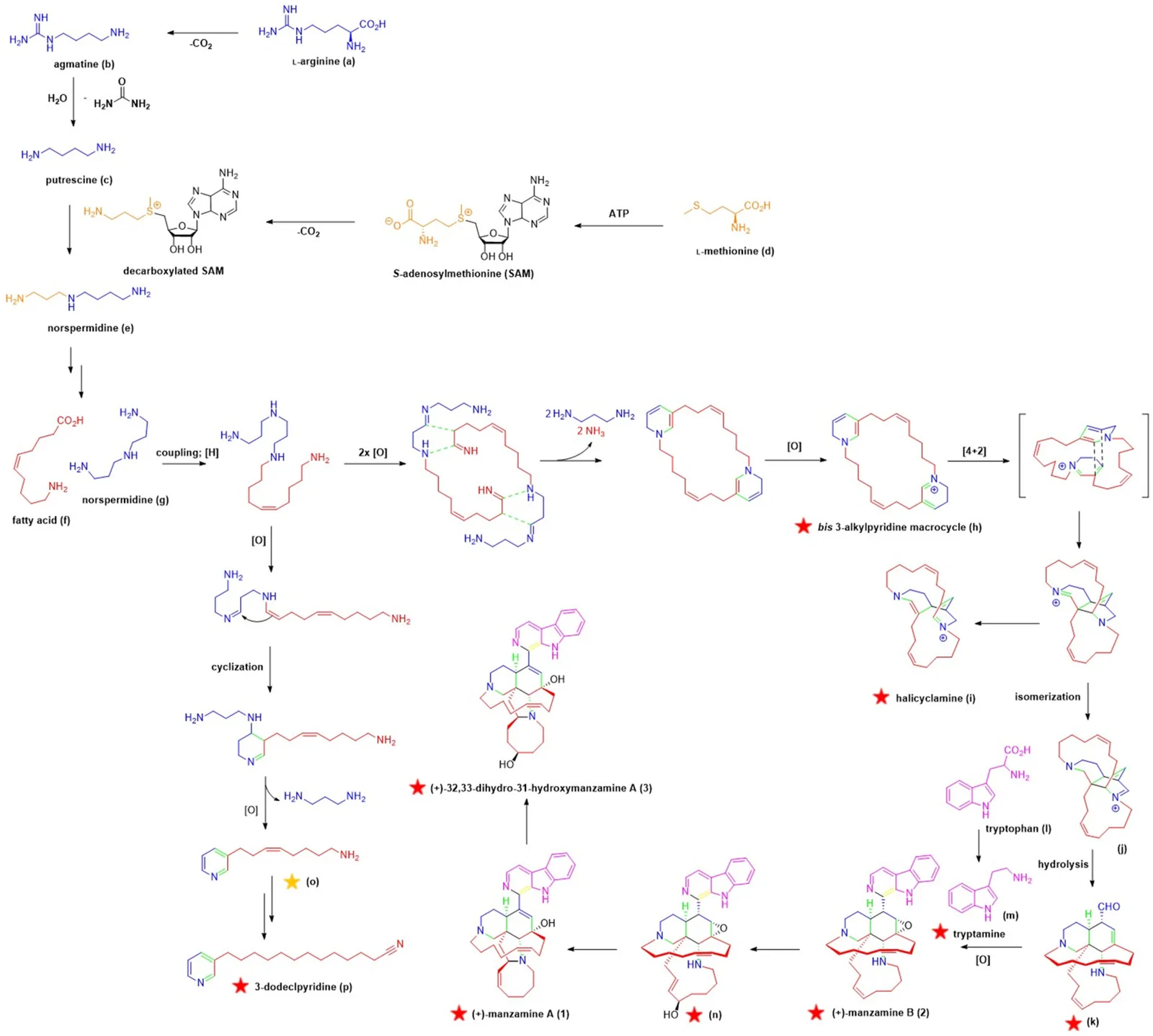
A unified plausible biosynthetic pathway of manzamine alkaloids and their precursors revealed by LC-HR-ESI-MS/MS analysis of organic layers obtained from solvent partitioning of extracts of *A. ingens* samples.

**Figure 3:**
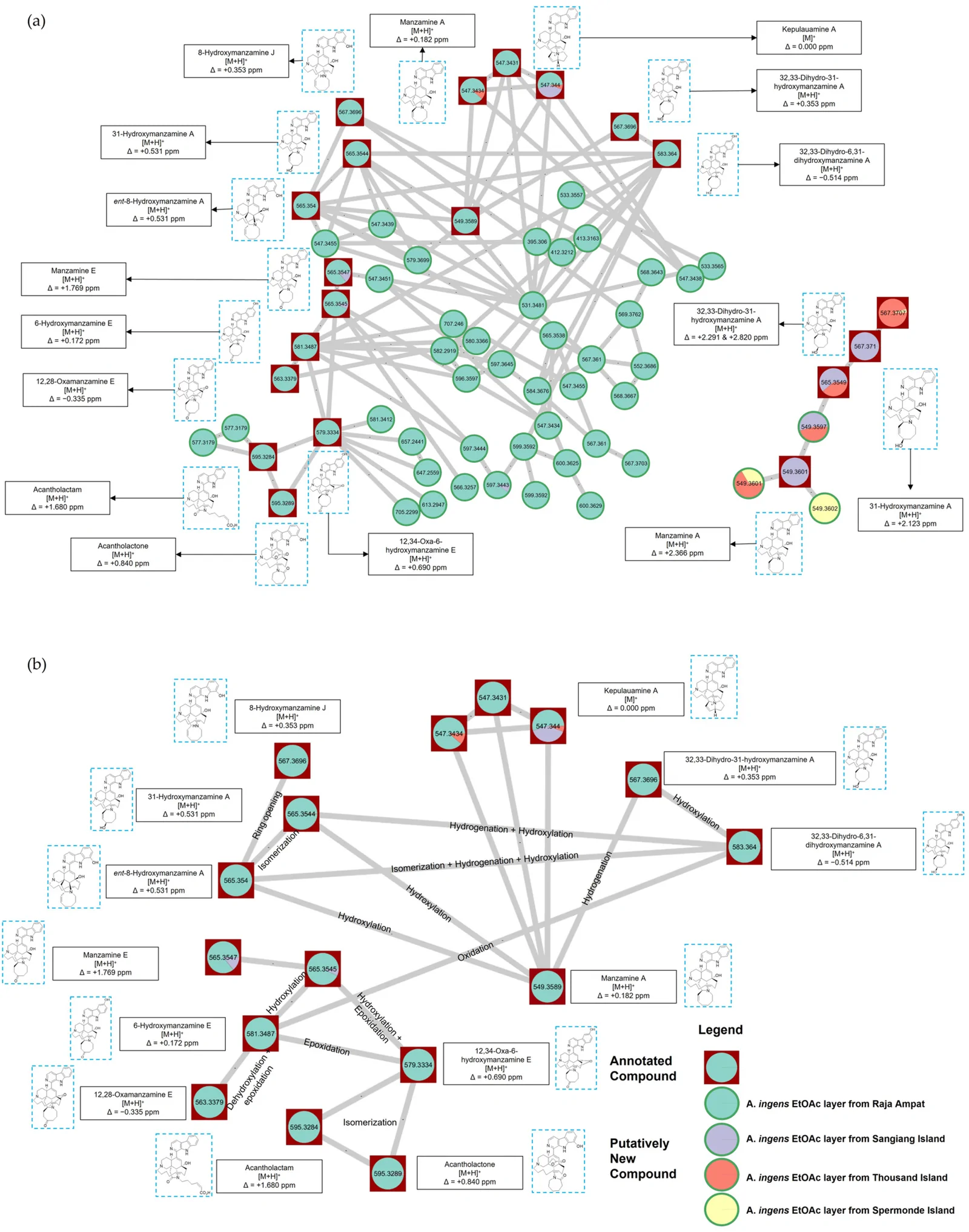
Molecular networking of EtOAc layers obtained from solvent partitioning of extracts prepared from four *A. ingens* samples showing molecular family related to manzamine alkaloids (**a**) and their possible chemical reactions involved in their transformations (**b**).

Through LC-HR-ESI-MS/MS and molecular networking analyses of the EtOAc layers, we were able to detect the presence of manzamine-related precursors and intermediates (Figure 2; indicated by red stars). Subsequently, a unified plausible biosynthetic pathway of manzamine A (**1**) and its related molecules, including **2** and **3**, is proposed (Figure 2 and Figure 3) [3,20,21,22]. The presence of (*Z*)-8-pyridin-3-yl)oct-5-en-1-aminium (**o**) was only observed by MS1 (yellow star) (Figure S4), while 3-alkyl pyridine represented by 3-dodecylpyridine (**p**) (Figure S5) and *bis* (3-alkylpyridine) macrocycle (**h**) (Figure S6) were observed in both MS1 and MS2, suggesting that fatty acid and norspermidine are likely involved in their biosyntheses. Subsequent intramolecular cycloaddition of *bis* (3-alkylpyridine) macrocycle (**h**) gave the halicyclamine skeleton (**i**) as shown possessing different MS2 fragmentation (Figure S7) compared with that of *bis* (3-alkylpyridine) macrocycle (**h**) (Figure S6). This experiment supported the presence of Diels-Alder products as in (**i**) and (**j**) which have been predicted by Baldwin and Whitehead in 1992 [23] and by Baldwin et al. in 1999 [24] on the biosynthetic studies of manzamine alkaloids. Further isomerization of halicyclamine followed by hydrolysis would provide the precursor of manzamine B (**k**) evidenced by LC-HR-ESI-MS/MS data (Figure S8). Tryptamine (**m**) was expected to be involved in the biosynthesis of manzamine B (**2**) (Figure S10) through a Pictet–Spengler reaction. The presence of tryptamine **(m**) previously detected by feeding experiment [4] is confirmed by LC-HR-ESI-MS/MS experiment in this study (Figure S9). Hydroxylation of manzamine B (**2**) followed by elimination would give manzamine A (**1**) (Figure S12) via its precursor (**n**) (Figure S11). Finally, (+)-32,33-dihydro-31-hydroxymanzamine A (**3**) (Figure S13) was detected as a modified natural product derived from manzamine A (**1**).

Additional related alkaloids, based on structural modification of manzamine A (**1**), were observed through the analysis of molecular networking (Figure 3a). At least 13 related molecules were detected, including 8-hydroxymanzamine J, 31-hydroxymanzamine A, *ent*-8-hydroxymanzamine A, manzamine E, 6-hydroxymanzamine E, 12,28-oxamanzamine E, acantholactam, acantholactone, 12,34-oxa-6-hydroxymanzamine E, 32,33-dihydro-31-hydroxymanzamine A, 32,33-dihydro-6,31-dihydroxymanzamine A, kepulauamine A, and 31-hydroxymanzamine A. In Figure 3b, the plausible biosynthetic relationship between these natural products is shown. Several chemical reactions are expected to be involved in their transformations, such as hydroxylation, dehydroxylation, epoxidation, hydrogenation, oxidation, isomerization, and ring opening. We have also annotated 34 possible known molecules and 267 putatively new molecules from LC-HR-ESI-MS/MS (Tables S1–S4, Figures S15–S18) and molecular networking analysis using Marinlit database (Figure S14).

**Figure 4:**
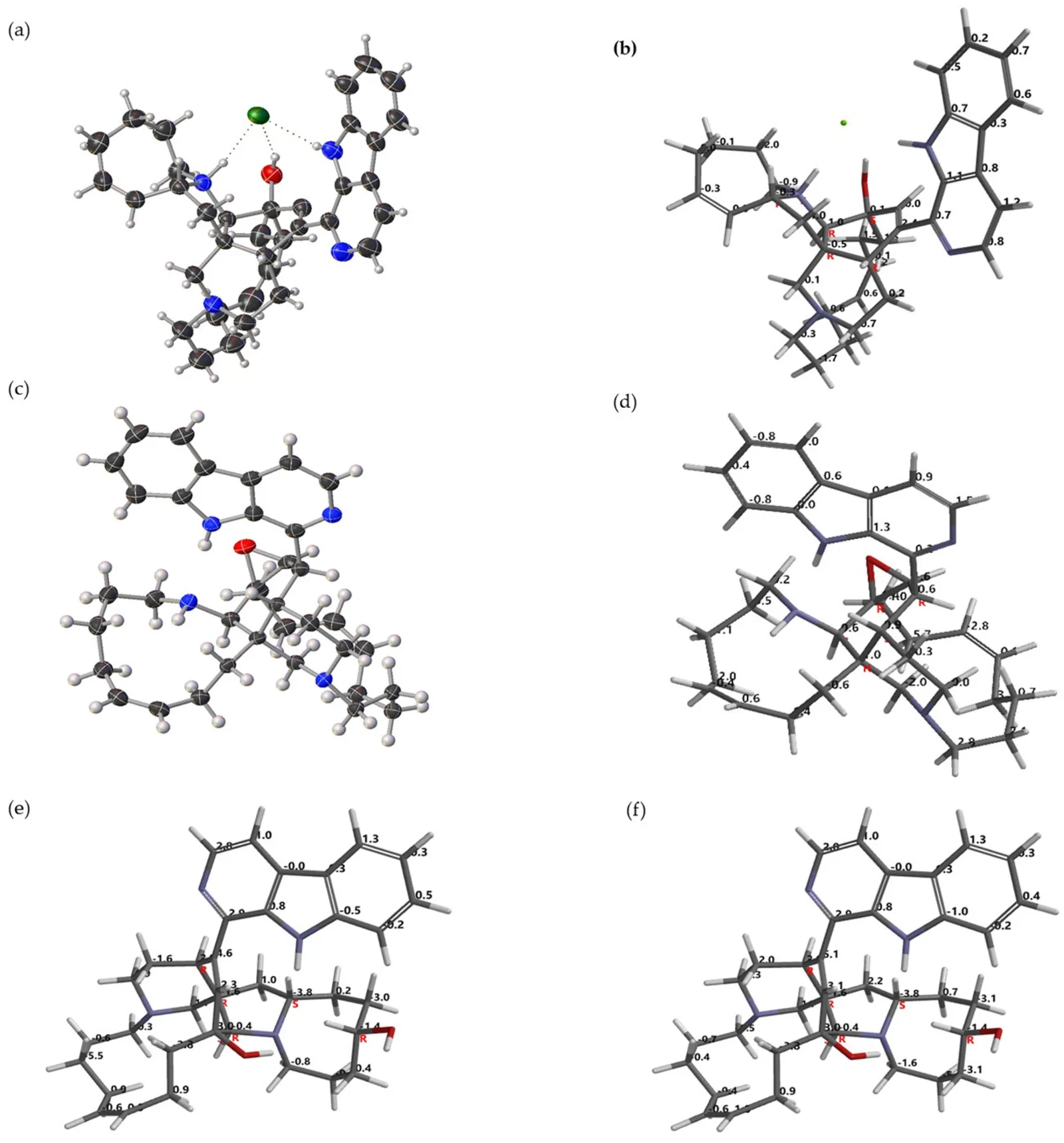
ORTEP drawing of manzamine A HCl (**1**) with displacement ellipsoids drawn at the 50% probability level (**a**); structure of 12S*, 24R*, 25R*, 26R*, 34R*-**1** obtained from the NMR calculation study (DP4 of 100%, RMSE of 0.94 ppm) (**b**); ORTEP drawing of manzamine B (**2**) with displacement ellipsoids drawn at the 50% probability level (**c**); structure of 10R*, 11S*, 12R*, 24S*, 25R*, 26R*-**2** obtained from the NMR calculation study (DP4 of 100%, RMSE of 1.58 ppm) (**d**); structure of 12S*, 24R*, 25R*, 26R*, 31R*, 34S*-**3** obtained from the NMR calculation study (DP4 = 99.3%, RMSE = 2.16 ppm) (**e**); structure of 12S*, 24R*, 25R*, 26R*, 31R*, 34S*-**3** with better assignment by NMR calculation study resulted in DP4 of 100% and RMSE of 1.99 ppm is shown (**f**).

From the sponge samples collected from the Thousand Islands, three alkaloids, including (+)-manzamine A hydrochloride (**1**), (+)-manzamine B (**2**), and (+)-32,33-dihydro-31-hydroxymanzamine A (**3**), were obtained. Their structures were confirmed based on NMR, HRMS, optical rotation, and X-ray analysis. The NMR assignments of **1**–**3** were determined on the basis of 1D (^1^H, ^13^C, DEPT) and 2D NMR (COSY, HSQC, HMBC) as well as comparison with the literature data. The stereostructures of **1** and **2** were analyzed using single crystal X-ray crystallography and specific optical rotation. To validate a new computational method for stereochemical determination, all possible stereoisomers except for enantiomers of **1**–**3** were calculated for their theoretical ^13^C NMR data employing DFT-based calculation and compared with the experimental data. The statistical decision was mainly made by using DP4 and RMSE. Finally, the most possible stereoisomer for each molecule was proposed in Figure 4 together with the three-dimensional-structure comparison between NMR calculation and the X-ray data for **1** and **2**.

Molecular shape, symmetry and stereogenicity of **1** and **2** were elucidated using X-ray analysis and NMR calculations. Both strategies are in a good agreement for stereostructure determination of **1** and **2** as shown in Figure 4a–d. The most possible stereostructure (1/16 calculated stereoisomers) of **1** is 12S*, 24R*, 25R*, 26R*, 34R* with DP4 of 100% and RMSE of 0.94 ppm (Figure 4b), while for **2** (1/15 calculated stereoisomers) is 10R*, 11S*, 12R*, 24S*, 25R*, 26R* with DP4 of 100% and RMSE of 1.58 ppm (Figure 4d). Finally, the stereostructure of **3** (1/32 calculated stereoisomers) was established using the NMR-based calculation. The relative configuration of **3** is likely 12S*, 24R*, 25R*, 26R*, 31R*, 34S* with DP4 of 99.3% and RMSE of 2.16 ppm (Figure 4e). Both experiments are in agreement with X-ray analysis done by Rao et al. [25]. Moreover, NMR calculation suggested the better assignment of 12 CH_2_ (#17, 18, 19, 20, 22, 23, 28, 29, 30, 32, 33, 35), 4 CH (#3, 7, 15), C (#8a, 10), giving DP4 of 100% and RMSE of 1.99 ppm (Figure 4f). Therefore, the improved assignment by NMR calculation is currently proposed (Figure 4f). Crystals of **1** and **2** were submitted for X-ray analysis (Figure 4a,c). For **1**, the presence of β-carboline, 5-, 6-, 6-, 8-, and 13-membered rings is clearly shown. The piperidine and cyclohexene rings adopted chair and boat conformation, respectively, while pyrrolidinium ring was an envelope. The conformation of the eight-membered ring containing *Z*-olefin was an envelope. The two six-membered rings bridged by a chain of nine carbon atoms make a 13-membered macrocycle perfectly ordered and rigid. The chloride ion was held within the molecule by hydrogen bonding with two NH and one OH groups. Consequently, the presence C-^+^NHR_2_ bond length was longer than usual bond lengths (observed: 1.522 Å). For **2**, the presence of epoxide was intact despite the favorable *trans*-disposed vicinal hydrogen substituent, the elimination of which undoubtedly accounts for the allylic alcohol grouping in **1**. Conversely, the reductive scission of the C-N bond common to the 5- and 8-membered rings in **1** is formally responsible for the new 11-membered ring in **2**.

All molecules, **1**–**3**, were evaluated for their biological activities using brine shrimp (*Artemia salina*), human immortal kidney HEK293T cells, Gram-positive *Staphylococcus aureus* and Gram-negative *Escherichia coli*, and α-glucosidase enzyme. As shown in Table 1, manzamine A.HCl (**1**) was the most potent molecule against *A. salina* (LC_50_ value of 0.041 ± 0.012 µM). The toxic effect of **1** to *A. salina* was about 2–40-fold more potent than the positive control molecules, including swinholide A, latrunculin A, laulimalide, paclitaxel, and doxorubicin (Table 1). Confirmation of the cytotoxicity against HEK293T cells indicated that **1** showed the most potent cytotoxicity (IC_50_ value of 0.599 ± 0.057 µM) among the isolated molecules. HEK293T cells are commonly used as a human cell line model in early cytotoxicity and safety evaluation of small molecules. The structure–activity relationship (SAR) suggested that the presence of azocine ring (*Z*-olefin at C32–C33) in **1** is important for toxicity [26].

In the antibacterial assay, only compound **1** showed a clear zone inhibition of 1.98 ± 0.06 mm (100 µg/disk) against *Staphylococcus aureus*, while no zone inhibition was observed for *Escherichia coli*, indicating **1** has weak antibacterial activity. Compound **1** also showed weak α-glucosidase inhibitory activity with IC_50_ of 1.04 ± 0.009 mM, while positive control, acarbose, showed IC_50_ value of 0.51 ± 0.06 µM. Compounds **2** and **3** showed no inhibition at 100 µg/disk against *S. aureus* and *E. coli.* As positive control, oxacillin (5 µg/disks) gave a clear zone inhibition of 26.58 ± 0.14 mm against *S. aureus*. Gentamicin (50 µg/disks) gave a clear zone inhibition of 19.31 ± 0.52 mm for *E. coli,* while chloramphenicol (50 µg/disks) gave a clear zone inhibition of 19.97 ± 0.27 mm for *S. aureus* and 14.73 ± 1.28 mm for *E. coli*.

Although numerous biological activities have been reported for manzamine A, Samoylenko and their co-workers concluded that compound **1** exhibits greater potency than its free base form across a wide range of bioassays. For example, manzamine A salt (**1**) possessed antibacterial activity against methicillin-resistant *Staphylococcus aureus* (IC_50_ value of 0.19 ± 0.001 µg/mL; MIC at 20 µg/mL) [27]. Compound **1** also exhibited cytotoxicity against Vero cell (IC_50_ value of 0.43–1.2 µg/mL). The antibacterial manzamine B (**2**) was reported to show weak or no activity against *S. aureus* (MIC at >100 ng/mL), *B. subtilis* (MIC at 100 ng/mL), *Kocuria rhizophila* (MIC at 100 ng/mL), *Proteus hauseri* (MIC at >100 ng/mL), and *E. coli* (MIC at >100 ng/mL), while it was more active against *Salmonella enterica* (MIC at 50 ng/mL) [28]. Manzamine B (**2**) also showed biological activity against A549 (LC_50_ value of 6.7 µM) and K562 (LC_50_ value of 9.1 µM) cells [28]. In the case of 32,33-dihydro-31-hydroxymanzamine A (**3**), it was more active against *S. aureus* (MIC at 50 ng/mL) due to the different ionic forms of the two molecules, while no or weak activity against *E. coli* (MIC at >100 ng/mL) was observed [28]. Compound **3** possessed cytotoxicity against A549 (LC_50_ value of 8.2 µM) and K562 (LC_50_ value of 8.4 µM) cells [28]. The obtained results in this study were generally in agreement with those reported in the literature.

**Figure 5:**
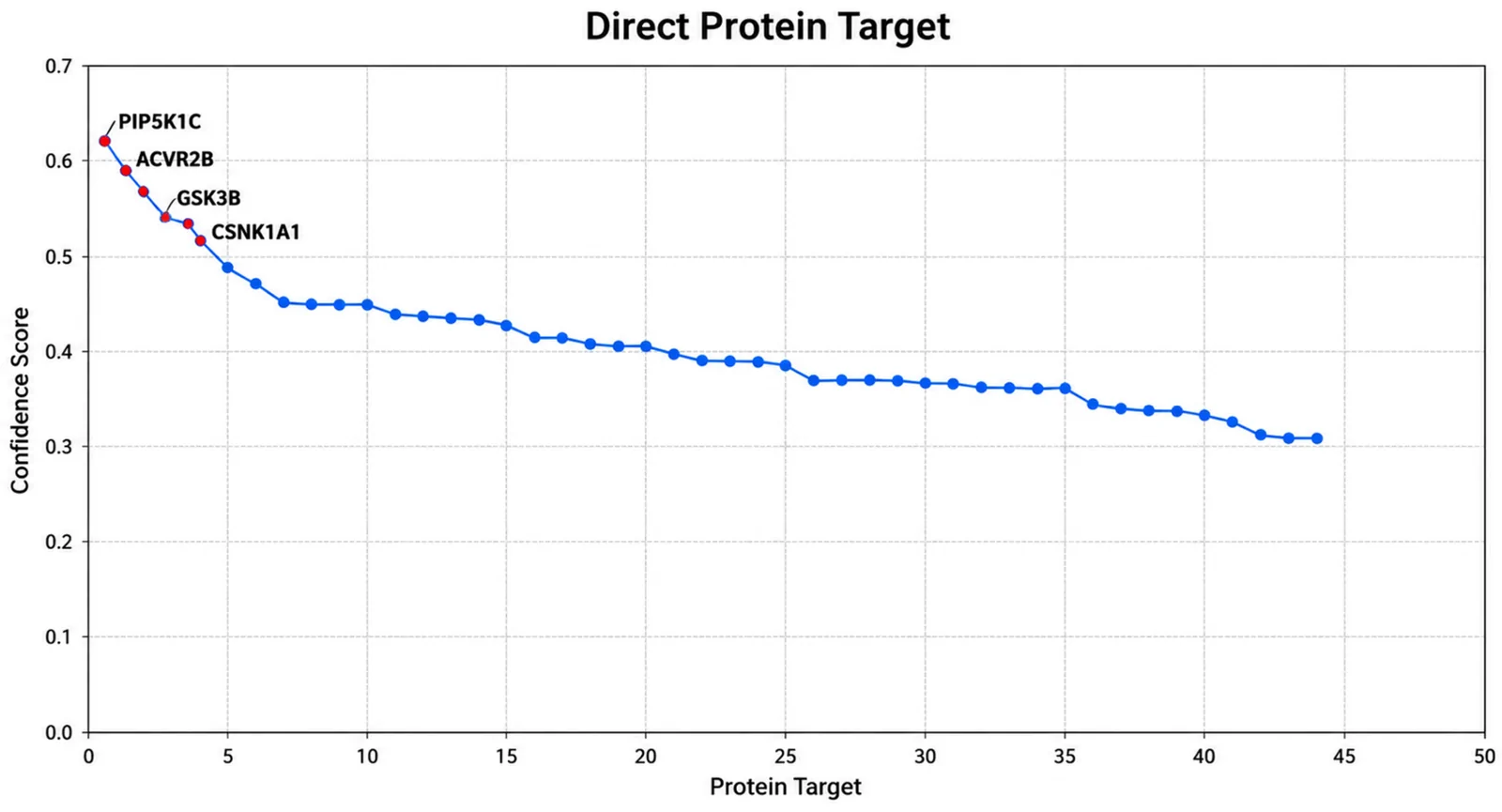
Forty-four potential protein targets of manzamines.

**Figure 6:**
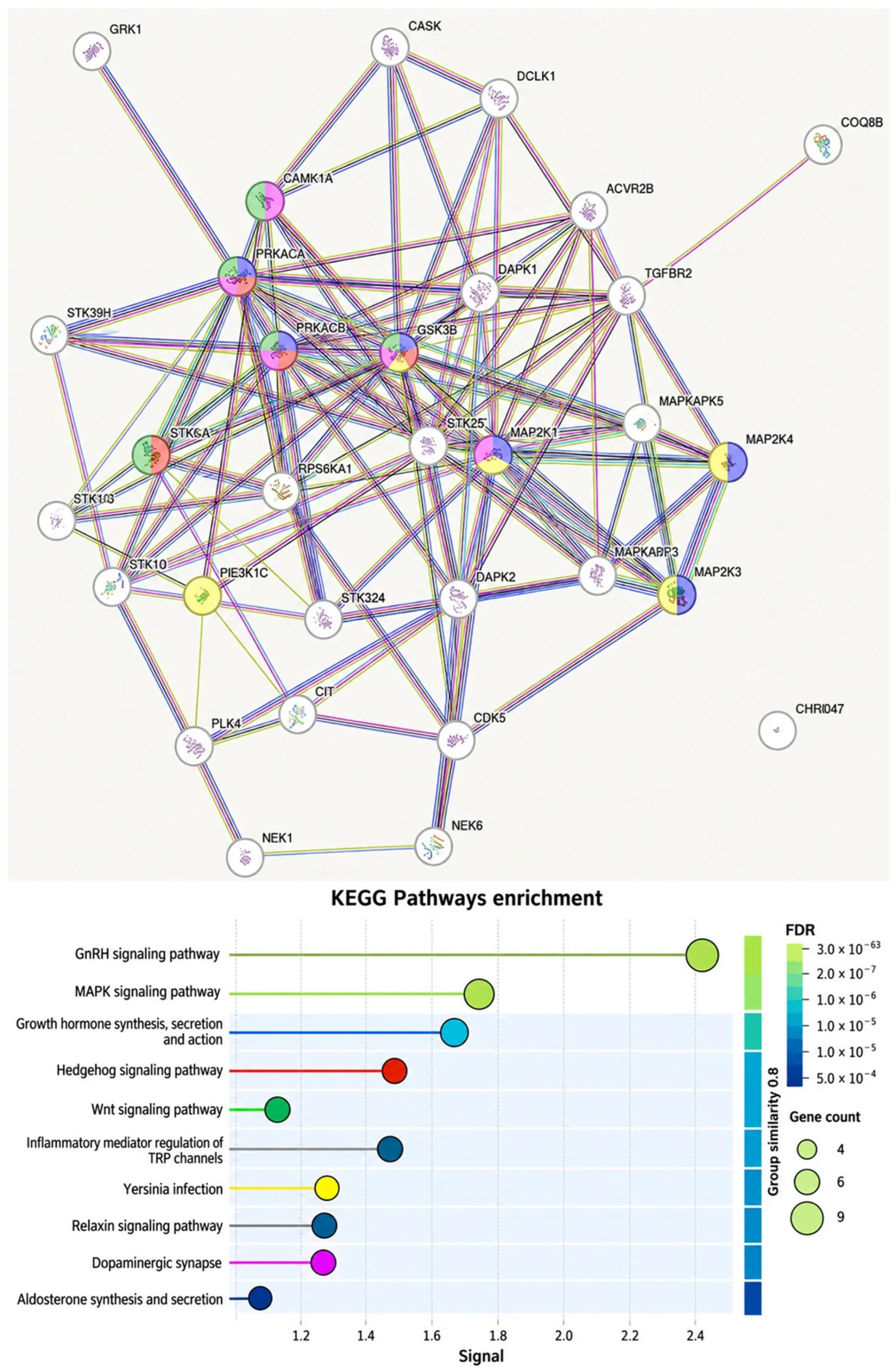
The Gene Ontology (GO) for targeted proteins by manzamines. Each color node indicates functionally related pathways grouped based on Jaccard similarity with a threshold of 0.8.

Target identification represents a critical early step in the drug discovery pipeline. The success of therapeutic development largely depends on selecting biologically relevant targets that are directly involved in disease progression. The importance of protein target identification can also be observed in the context of precision medicine. Identification of disease-specific protein targets enables researchers to design drugs with greater selectivity, thereby reducing off-target toxicity and adverse side effects. We found four potential hit molecular targets, including PIPK1C, ACVR2B, GSK-3β, and CSNK1A1, of the manzamines in this study (Figure 5). We further employed the Gene Ontology (GO) in enrichment analysis using string DB (protein–protein interaction) (Figure 6).

The Gene Ontology (GO) enrichment findings further support the potential role of GSK-3β as a promising therapeutic target in cancer research. GO analysis demonstrated that the identified targets were significantly associated with biological processes involved in cell proliferation, apoptosis regulation, signal transduction, and inflammatory responses, all of which are critical mechanisms underlying cancer initiation and progression. Among these proteins, GSK-3β appears to occupy a central regulatory position due to its involvement in multiple oncogenic signaling pathways, including the Wnt signaling pathway (Figure 6).

**Figure 7:**
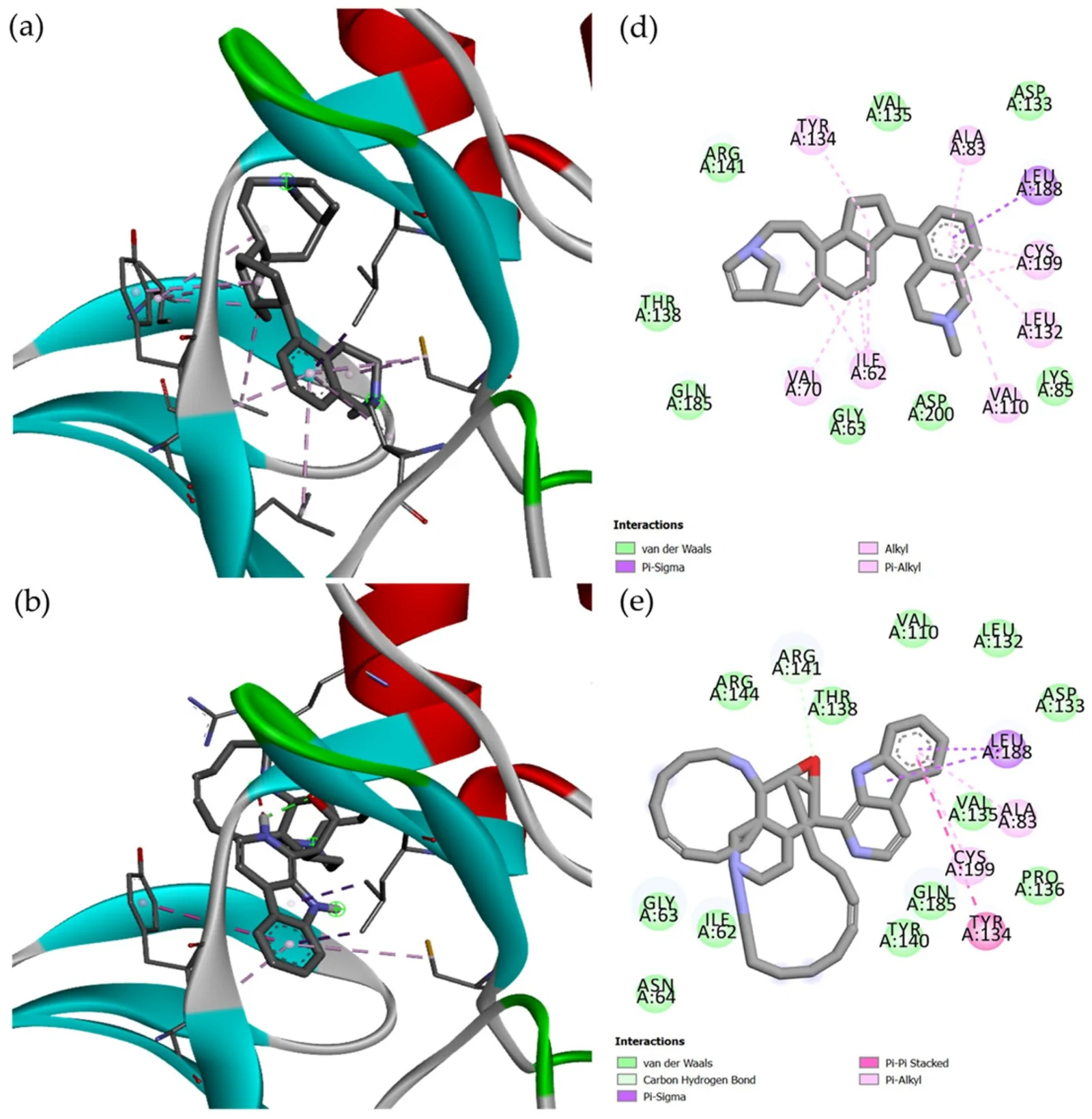

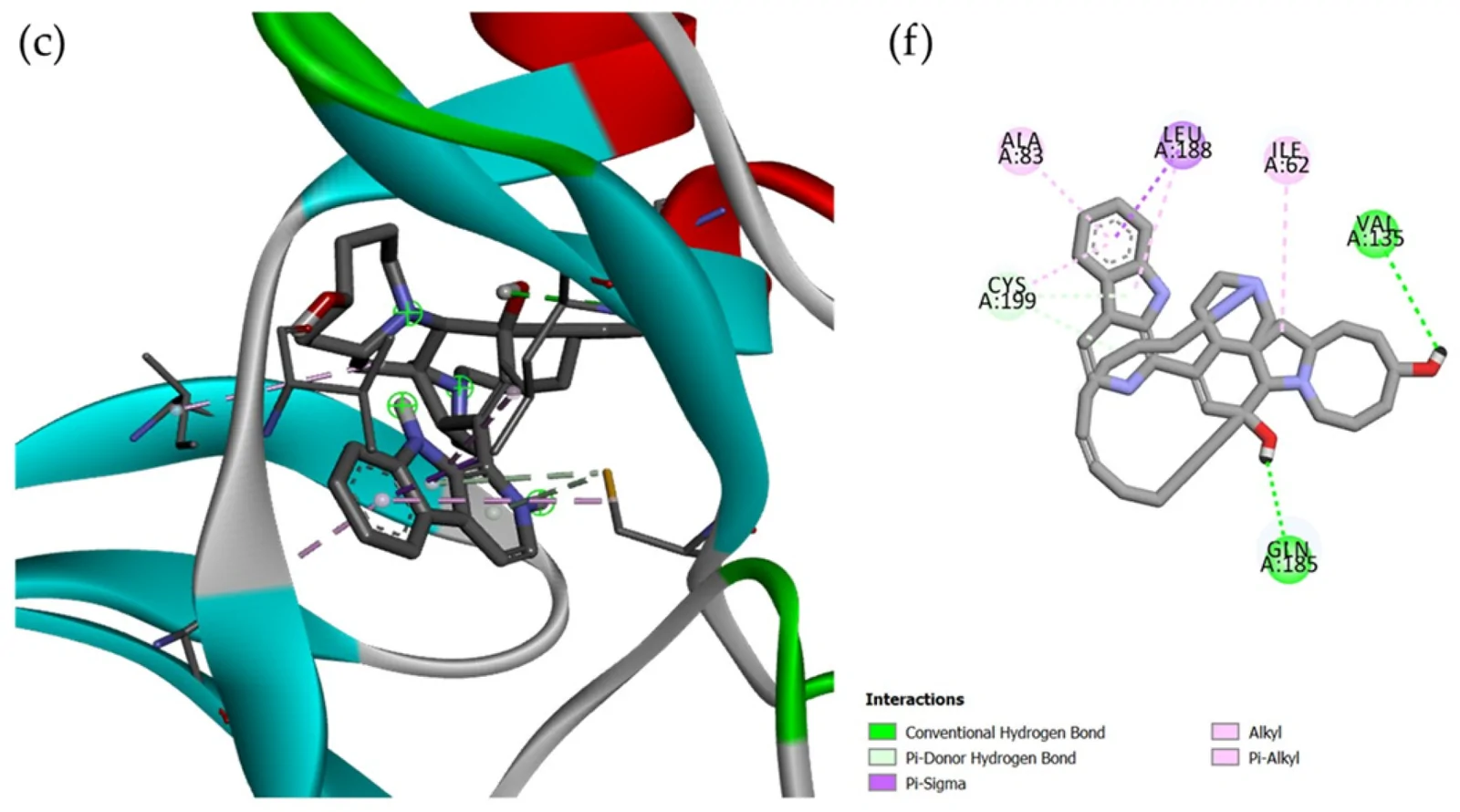
Molecular docking of manzamine A hydrochloride (**1**), manzamine B (**2**), and 32,33-dihydro-31-hydroxymanzamine A (**3**) with GSK-3β: 3D binding poses of **1** (**a**); **2** (**b**); **3** (**c**) within the ATP-binding pocket of GSK-3β. Corresponding 2D interaction diagrams are shown for **1** (**d**), **2** (**e**), and **3** (**f**).

The identification of GSK-3β through GO-based functional analysis provides additional evidence supporting its therapeutic relevance in anticancer drug development. Molecular docking also demonstrated that all three manzamine derivatives exhibited favorable binding toward GSK-3β (PDB ID: 7U31), with docking scores summarized in Table 2. Compound **3** exhibited the strongest binding affinity toward GSK-3β (−10.6 kcal/mol), followed by **2** (−10.4 kcal/mol) and **1** (−9.8 kcal/mol), whereas the native ligand showed a lower binding affinity of −7.6 kcal/mol. In molecular docking, binding affinity is commonly used to estimate the strength of ligand–protein interactions, where more negative values indicate stronger binding and greater complex stability [29]. In addition, these compounds were consistently positioned within the ATP-binding pocket and interacted with key catalytic-site residues, including Ala83, Lys85, and Asp200 (Figure 7). The docking result of (+)-**1** was in agreement with the reported GSK-3β inhibition in vitro and cell-based assay [12,13].

As shown in Table 2, results of molecular docking and cytotoxicity assays provide complementary but fundamentally different levels of biological information. Molecular docking predicts the potential interaction between a ligand and a predefined molecular target based on a computational scoring function, whereas cytotoxicity assays measure the integrated phenotypic response of living cells to compound exposure. Therefore, docking results should be interpreted as evidence supporting a potential molecular mechanism rather than direct evidence of cytotoxic potency. A favorable docking score may support the hypothesis that a compound can interact with the proposed target, but experimental target-binding or target-inhibition assays are required to establish target engagement. Likewise, cytotoxicity data should be interpreted at the cellular level and should not be directly equated with predicted docking affinity.

Furthermore, analysis of the docking poses revealed that complex formation was primarily stabilized through a combination of hydrogen-bond and hydrophobic interactions with residues located within the catalytic region of GSK-3β (Figure 7). Compound **1** interacted with several residues, including Ile62, Gly63, Val70, Ala83, Lys85, Val110, Leu132, Asp133, Tyr134, Val135, Thr138, Arg141, Gln185, Leu188, Cys199, and Asp200. Similar interaction patterns were observed for compounds **2** and **3**, which also occupied the catalytic cleft of the kinase. Notably, several residues identified in the present study, including Lys85, Asp133, Val135, Arg141, Gln185, and Asp200, have previously been recognized as critical determinants of ligand binding and stabilization within the GSK-3β active site [30]. The interaction of the manzamine derivatives with these residues therefore supports their accommodation within the canonical ATP-binding region and reinforces the predicted inhibitory potential of these compounds. The presence of hydroxyl-containing functional groups in **2** and **3** contributed to additional hydrogen-bond interactions, which may account for their slightly improved binding affinities relative to **1**.

Notably, all compounds occupied the same functional region as reported for GSK-3β inhibitors, suggesting a potential ATP-competitive mode of inhibition. Interactions involving residues associated with ATP recognition and catalytic activity further support the ability of these marine alkaloids to modulate GSK-3β function [31]. Given the central role of GSK-3β in tau hyperphosphorylation and neurodegeneration [32], these findings provide a molecular rationale for the neuroprotective potential previously reported for manzamine derivatives.

The interaction maps further indicated that all compounds were positioned within the same functional region of the kinase, overlapping with the ATP-binding site occupied by known GSK-3β inhibitors. Such binding behavior suggests a potential ATP-competitive mechanism of action. Notably, residues including Lys85 and Asp200, which play crucial roles in kinase catalytic activity and ATP recognition, participated in ligand stabilization [33], supporting the hypothesis that these marine alkaloids may interfere with GSK-3β-mediated phosphorylation processes.

The observed interactions may contribute to kinase inhibition by interfering with ATP binding and subsequent phosphorylation events. Given the involvement of GSK-3β in multiple signaling pathways associated with cancer progression, including the Wnt/β-catenin, PI3K/Akt, and NF-κB pathways [32], modulation of GSK-3β activity has emerged as a promising therapeutic strategy in oncology. Therefore, the favorable binding affinities and interaction profiles observed for compounds **1**–**3** provide a plausible molecular basis for their cytotoxic activities and support the potential contribution of GSK-3β as a molecular target underlying the anticancer properties of manzamine alkaloids.

## 3. Materials and Methods

### 3.1. General Methods

Optical rotations of isolated molecules were obtained with an Anton Paar MCP 5100 digital polarimeter (Anton Paar GmbH, Graz, Austria). NMR spectra were recorded on a 500 MHz Bruker Avance III spectrometer (Bruker Coorp., Billerica, MA, USA) or a 500 MHz Varian (Varian Assoc., Palo Alto, CA, USA). The chemical shifts were expressed in *δ* (ppm) and the coupling constants (*J*) in Hz. Chemical shifts were referenced to tetramethylsilane (TMS) or CDCl_3_ signals. MS spectra were recorded on a Waters Acquity Xevo G2-S ESI-Q-TOF (Waters Coorp., Milford, MA, USA), HR-ESI-TOF-MS JEOL T100LP (JEOL Ltd., Tokyo, Japan) or Hitachi M-2500 instrument (Hitachi Ltd., Tokyo, Japan). UV spectra were obtained on a Shimadzu Pharmaspec 1700 spectrophotometer (Shimadzu Coorp., Kyoto, Japan). X-ray analysis was performed on a Rigaku AFC10 goniometer equipped with a Saturn 724+ detector (Rigaku Coorp., Tokyo, Japan). High-performance liquid chromatography (HPLC) separations were carried out on a Hitachi L-6000 pump (Hitachi Ltd., Tokyo, Japan) fitted with Shodex RI-101 refractive index (Shoko Science Co., Ltd., Tokyo, Japan) and SPD-20A Shimadzu UV detectors (Shimadzu Coorp., Kyoto, Japan), or a Shimadzu HPLC (Shimadzu Coorp., Kyoto, Japan) with Prominence LC20AD, DGU-20A5, SPD-20A or a Shimadzu HPLC (Shimadzu Coorp., Kyoto, Japan) with SPD-10A UV-Vis detector (Shimadzu Coorp., Kyoto, Japan), LC-10AT pump (Shimadzu Coorp., Kyoto, Japan), and SCL-10A system controller (Shimadzu Coorp., Kyoto, Japan). A Cosmosil 5SL-II-MS (normal silica, 20 mm of I.D. × 250) column was used for HPLC. Analytical thin-layer chromatography (TLC) was performed on Merck silica gel 60 F254 plates (Merck KGaA, Darmstadt, Germany) and visualized with Dragendorff and UV. All solvents used were reagent-grade.

### 3.2. Animal Material

Samples of the marine sponge, *Acanthostrongylophora ingens*, were collected from Waigeo Island, Raja Ampat, in 2022 (S 00°28.528′ E 130°50.143′, −22 m), the Thousand Islands in 2016 and 2019 (near Pramuka Island, S 5°44′45.0′ E 106°36′50.7″, −10 to −15 m), Pajenekang Island and Spermonde Archipelago in 2024 (S 4°58.340′ E 119°19.337′, −12 m) in 2024, and Tanjung Bajo and Sangiang Island in 2025 (S 5°56′13.263″ E 105°51′23.084″, −20 m) by hand, using SCUBA. Upon collection, specimens were immediately frozen at −20 °C or stored in EtOH 96% and transported to IPB University. Subsamples of the specimens were identified as *A. ingens* by Prof. Dr. Nicole J. de Voogd. Sponge skeletal architecture and spicule composition were examined using a Leica DM5500 light microscope (Leica Microsystem, Wetzlar, Germany). Hand-cut sections of the ectosome and choanosome were prepared to study skeletal architecture, while spicules were isolated by dissolving tissue in dilute sodium hypochlorite (3–5%), followed by washing with distilled water and ethanol. Slides were analyzed and photographed using a fixed camera system. For each spicule type, 25 fully developed spicules were measured to determine minimum, mean, and maximum length and width. The sponge forms thick, submassive structures composed of single or multiple fused tubes. Its consistency is very friable. In life, the external surface is reddish-brown, while the interior is yellowish. The ectosomal skeleton consists of an irregular to subrectangular reticulation of strongyles with sparse spongin and numerous loose spicules. The choanosomal skeleton is composed of multispicular longitudinal tracts (70–120 µm in diameter) interconnected by irregular meshes with only small amounts of spongin. The spicules are smooth, with slightly curved strongyles measuring 67–120–161 µm (min–mean–max) in length and 3–5–8 µm (min–mean–max) in width.

### 3.3. Extraction and Solvent Partitioning of Sponge-Derived Extracts

A portion of each *A. ingens* specimen (about 10 g) collected from the Thousand Islands in 2019, Raja Ampat in 2022, Pajenekang Island in 2024, and Sangiang Island in 2025 was extracted exhaustively using MeOH (4 × 10 mL) and concentrated in vacuo to provide MeOH extracts (2.1–2.4 g). Each MeOH extract was then partitioned between EtOAc (5 mL) and H_2_O (2.5 mL) three times to give EtOAc (0.6–0.9 g) and H_2_O layers.

### 3.4. LC-HR-ESI-MS/MS Analysis

The samples were analyzed using a UHPLC Vanquish Tandem Q Exative plus orbitrap HRMS Thermo Scientific (Thermo Fischer, Waltham, MA, USA). The Accucore C18 stationary phase (2.1 mm × 100 mm, 1.5 µm, Thermo Scientific) was used, while the mobile phase was a mixture of 100% H_2_O + 0.1% formic acid (A) and 100% MeCN + 0.1% formic acid (B) with gradient elution from 5% B (0–1 min) to 5–95% B (1–25 min), 95% B (25–28 min), and 5% B (28–33 min). The column temperature was maintained at 30 °C, and the injection volume was 2 µL. The conditions used for HRMS were described as in [34]. The LC-HR-ESI-MS/MS data were processed using FreeStyle™ 1.8 SP2 QF1 software. Molecular formula of notable MS1 peak from each peak in LC chromatogram was analyzed using Freestyle’s elemental composition prediction feature. Prediction results are limited to formula composed of C, H, N, O with absolute mass error below 5 ppm from its theoretical mass. Highest ranked prediction based on several parameters (S fit, RDB, matched isomers, MS coverage and pattern coverage) is chosen to be the most probable formula for the corresponding mass peak. Information extracted from each chromatogram is then crosschecked with the Marinlit database to identify known/unknown compound.

### 3.5. MZmine 4.9.0 Data Pre-Processing, Feature-Based Molecular Networking, and Chemoinformatic Database Analyses

The data pre-processing using Mzmine 4.9.0, feature-based molecular networking, and chemoinformatics database analyses were performed based on the literature [26]. For network creation, both the parent mass and fragment ion tolerance were set to 0.02 Da. Edges were filtered to have a cosine score above 0.7, maximum shift between precursor = 500 Da, and at least six matched peaks. Furthermore, the GNPS link can be seen as in https://gnps.ucsd.edu/ProteoSAFe/status.jsp?task=ceefaabb19ed4903b4cf7241df335415 (30 March 2026) with ID: ceefaabb19ed4903b4cf7241df335415.

### 3.6. Isolation and Purification of Manzamine Alkaloids

The fresh sponge specimen collected from the Thousand Islands in 2019 (wet weight of 592 g) stored in EtOH of 96% was extracted with EtOH of 96% (4 × 1 L). The combined extract was concentrated using vacuum rotary evaporator. The resulting residue (24.5 g) was partitioned between EtOAc (200 mL) and H_2_O (50 mL) 4 times. The EtOAc layer was then concentrated and the toxicity of its extract (4.3 g) was evaluated against the larvae *A. salina*. The cytotoxic lipophilic EtOAc layer was separated using a Si gel 60 column (±80 g) by eluting stepwise with CHCl_3_ (100% *v*/*v*, 200 mL), CHCl_3_–MeOH (90%: 10% *v*/*v*; 80%: 20% *v*/*v*; 60%: 40% *v*/*v*; 40%: 60% *v*/*v*, 100 mL each), and MeOH (100% *v*/*v*, >100 mL) to give 13 subfractions (**A** = 0.008 g; **B** = 0.0868 g; **C** = 1.0222 g; **D** = 0.064 g; **E** = 0.0408 g; **F** = 0.0141 g; **G** = 1.1058 g; **H** = 0.0896 g; **I** = 0.2420 g; **J** = 0.6718 g; **K** = 0.0580 g; **L** = 0.3075 g; **M** = 10.0986 g). A portion of **C** was recrystallized using CHCl_3_–MeCN to give a pure compound **1** (57.6 mg) with a high quality of crystal. Another marine sponge *A. ingens* (400 g) collected from the Thousand Islands in 2016 was extracted with EtOH of 96% (4 × 1 L). The EtOH extract (13.4 g) was then partitioned between hexane (100 mL) and 90% *v*/*v* MeOH–H_2_O aqueous (50 mL) 4 times. The latter was then partitioned between CH_2_Cl_2_ (100 mL) and 50% *v*/*v* MeOH–H_2_O aqueous (50 mL) 4 times. Finally, the 50% *v*/*v* MeOH–H_2_O aqueous was partitioned between BuOH (100 mL) and H_2_O (50 mL) 4 times. The CH_2_Cl_2_ layer (1.47 g), toxic against zebrafish embryos, was purified using silica gel 60 column (±60 g) by eluting stepwise with CH_2_Cl_2_ (100% *v*/*v*, 200 mL), CH_2_Cl_2_–EtOAc (60%: 40% *v*/*v*; 40%: 60% *v*/*v*; 20%: 80% *v*/*v*; 10%: 90% *v*/*v*, 100 mL each), EtOAc (100% *v*/*v*, 100 mL), and MeOH (100% *v*/*v* >100 mL) to give five subfractions (**A** = 0.0011 g; **B** = 0.1408 g; **C** = 0.0717 g; **D** = 0.0336 g; **E** = 0.5538 g). A portion of cytotoxic subfraction **C** was then recrystallized using CH_2_Cl_2_–MeOH to give compound **3** (1.5 mg). Cytotoxic subfraction **E** was also purified using normal silica gel 60 (0.8 g) eluted with CHCl_3_ (100% *v*/*v*), CHCl_3_–MeOH (88.9%: 11.1% *v*/*v*), and MeOH (100% *v*/*v*) to give 8 subfractions (**E1** = 2.5 mg; **E2** = 110.1 mg; **E3** = 90.6 mg; **E4** = 87.4 mg; **E5** = 14.8 mg; **E6** = 57.2 mg; **E7** = 33.1 mg; **E8** = 4.8 mg). The subfraction **E2** was finally purified using HPLC normal Si eluted with CHCl_3_–MeOH (90%: 10% *v*/*v*) to give compound **2** (45.9 mg).

#### 3.6.1. (+)-Manzamine A Hydrochloride (**1**)

Colorless crystal (CHCl_3_–MeCN), with *R_f_* of 0.89 (CHCl_3_–MeOH 9/1, normal silica, positive Dragendorff reagent); [α]_D_^25^ = +53.1° (*c*, 0.05 CHCl_3_); and lit [α]_D_^20^ = +50° (*c*, 0.28 CHCl_3_) [1], ^1^H and ^13^C-NMR were identical with those reported in [1]. HR-ESI-TOF-MS has a *m*/*z* of 547.3380 [M-HCl-H]^−^ (theoretical mass *m*/*z* of 547.3442, calcd for C_36_H_43_N_4_O^−^, Δ −6.2 mmu). ^1^H and ^13^C NMR as well as HR-ESI-TOF-MS spectra of **1** together with its TLC profile were shown in Figure S1.

#### 3.6.2. (+)-Manzamine B (**2**)

Colorless crystal (CH_2_Cl_2_–MeOH), with *R_f_* of 0.73 (CHCl_3_–MeOH 9/1, normal silica, positive Dragendorff reagent); [α]_D_^25^ = +94.7° (*c*, 0.03 CHCl_3_); and lit [α]_D_^20^ = +89° (*c*, 1.8 CHCl_3_) [35], ^1^H and ^13^C-NMR were identical with those reported in [35]; HR-ESI-TOF-MS has a *m*/*z* of 551.3764 [M + H]^+^ (theoretical mass *m*/*z* of 551.3744, calcd for C_36_H_47_N_4_O^+^, Δ +2.0 mmu). ^1^H and ^13^C NMR as well as HR-ESI-TOF-MS spectra of **2** together with its TLC profile were shown in Figure S2.

#### 3.6.3. (+)-32,33-Dihydro-31-Hydroxymanzamine A (**3**)

Colorless oil, with *R_f_* of 0.78 (CHCl_3_–MeOH 9/1, normal silica, positive Dragendorff reagent); [α]_D_^26^ = +40.0° (*c*, 0.4 CHCl_3_); and lit [α]_D_^25^ = +34.44° (*c*, 0.9 CHCl_3_) [25], ^1^H and ^13^C-NMR were similar to those reported in [25]. ^1^H and ^13^C NMR spectra of **3** were shown in Figure S3.

### 3.7. X-Ray Crystallographic Analysis

Single crystals of C_36_H_44_ClN_4_O (**1**) and C_36_H_46_N_4_O (**2**) were supplied. A suitable crystal was selected and mounted on Rigaku Saturn 724 Plus with AFC10 (Rigaku Coorp., Tokyo, Japan). The crystal was kept at 293 and 93 K during data collection. Using Olex2 [36], the structure was solved with the SHELXT [37] structure solution program using direct methods and refined with the SHELXL [38] refinement package using least squares minimization.

Crystal data for **1**, C_36_H_44_ClN_4_O (*M* =584.20 g/mol), include: orthorhombic, space group P2_1_2_1_2_1_ (no. 19), *a* = 12.9863 (2) Å, *b* = 15.2744 (3) Å, *c* = 15.8922 (3) Å, *V* = 3152.34 (10) Å^3^, *Z* = 4, *T* = 293 (2) K, μ (CuKα) = 1.332 mm^−1^, and *D_calc_* = 1.231 g/cm^3^. A total of 35,947 reflections were measured (8.028° ≤ 2Θ ≤ 136.368°), where 5758 of them were unique (*R*_int_ = 0.0420, R_sigma_ = 0.0315) and used in all calculations. The final *R*_1_ was 0.0378 (I > 2σ(I)) and *wR*_2_ was 0.0967 (all data). The final Flack parameter was 0.080 ± 0.005, indicating that the present absolute structure is correct. The crystal data of **1** was deposited in Cambridge Crystallographic Data Center (CCDC) with a deposition number of 2580282.

Crystal data for **2**, C_36_H_46_N_4_O (M = 550.77 g/mol), include: orthorhombic, space group *P*2_1_2_1_2_1_ (no. 19), *a* = 9.2750 (3) Å, *b* = 17.6429 (6) Å, *c* = 18.0301 (6) Å, *V* = 2950.41 (17) Å^3^, *Z* = 4, *T* = 93 K, μ (CuKα) = 0.578 mm^−1^, and *D*_calc_ = 1.240 g/cm^3^. A total of 5389 reflections were measured (2θ_max_ = 136.62°), where 374 of them were unique (*R*_int_ = 0.0506) and used in all calculations. The final *R*_1_ was 0.0357 (*F*_2_ > 2.0σ(*F*_2_)) and *wR*_2_ was 0.0784 (all data). The final Flack parameter was 0.09, indicating that the present absolute structure is correct. The crystal data of **2** was deposited in CCDC with a deposition number of 2580283.

### 3.8. DFT NMR Calculation

DFT NMR calculation was performed using Spartan ’24 software [17]. As reported before [18], the calculation study consisted of six consecutive steps of optimization: (1) systematic conformational search with the Merck Molecular Force Field (MMFF) molecular mechanic model and removal of conformers with energies larger than 40 kJ/mol from its global minimum (maximum 200 conformers); (2) energy correction of conformers with machine learning model “Corrected MMFF” and elimination of conformers with energy higher than 20 kJ/mol from its global minimum (maximum 100 conformers); (3) geometry optimization with the HF/3-21G model and removal of duplicate conformers and those with energies larger than 30 kJ/mol from that of the global minimum (maximum 100 conformers); (4) energy optimization with the ωB97X-D/6-31G* model and removal of conformers with energies larger than 15 kJ/mol from that of the global minimum (maximum 50 conformers); (5) geometric optimization with the ωB97X-D/6-31G* model and removal of conformers with energies larger than 10 kJ/mol from that of the global minimum (maximum 50 conformers); and (6) energy optimization with the ωB97X-V/6-311 + G(2df,2p) [6-311G*] model (maximum 30 conformers). Finally, chemical shifts for all conformers within 10 kJ/mol from the global minimum were obtained using ωB97X-D/6-31G* GIAO theory and empirically corrected. NMR shifts in each conformer are Boltzmann-weighted based on energy computed previously and applied to the equilibrium conformer (global minimum). The DP4 analysis was conducted only for the carbon nucleus using Goodman’s parameters (13C: σ = 2.306 ppm and υ = 11.38).

### 3.9. Cytotoxicity Assays

#### 3.9.1. Brine Shrimp Lethality Assay

The cytotoxicity testing was conducted as previously reported [39]. Commercially available dried eggs of brine shrimp (*Artemia salina*) were incubated in artificial sea water at 25 °C for 24 h. A total of 10 hatched larvae were placed in a well of 24-well plates with brine (1 mL). The diluted test samples with a series concentration of 0.02; 0.03; 0.06; 0.13; 0.25; 0.50; 1.00; and 2.00 µg/mL were added to the well and the plates were kept at 25 °C. The mobility of the animals was observed for 24 and 48 h to count the number of live animals. An individual without any motion during the observation was regarded as dead. Triplicates were performed for each sample. DMSO was used as the negative control, while swinholide A, latrunculin A, laulimalide, doxorubicin, and paclitaxel were used as positive controls. The LC_50_ was calculated using IBM SPSS 22 software and expressed in µM together with its standard deviation (SD).

#### 3.9.2. In Vitro Cytotoxicity Assay Against HEK293T

In vitro cytotoxicity of test samples was determined against human embryonic kidney 293T (HEK293T) cells based on our previous report [19]. HEK293T cells are commonly used as a human cell line model in early cytotoxicity and safety screening of small molecules, including natural products. HEK293T cells were provided by the Primate Animal Study Research Center, IPB University (Bogor, Indonesia). The cytotoxic assay was performed on 96-well treated tissue culture plates. Cells were seeded in the wells (5000 cells in 100 µL of media containing RPMI1640, FBS, penicillin and streptomycin) and incubated for 24 h. The diluted test samples with concentrations of 0.03; 0.06; 0.13; 0.25; 0.50; 1.00; 5.00; and 10.00 µg/mL were then added. The plates were again incubated for 48 h. MTT was then added and the plates were incubated for 4 h at 37 °C. Formazan crystals formed were dissolved in EtOH and absorbances were read at λ = 595 nm. The result was analyzed using Prism 9 software (Graphpad) to obtain IC_50_ values with its standard deviation (SD).

#### 3.9.3. Agar-Plate Diffusion Assay

Gram-positive bacteria *Staphylococcus aureus* and Gram-negative bacteria *Escherichia coli* were used for biological evaluation of the three manzamine compounds (**1**–**3**). The bacteria were provided by the Tropical Biopharmaca Research Center and/or IPB culture collection from IPB University. Antibacterial assay was performed using agar-plate diffusion assay [19] with a 100 µg/disks concentration of each compound. A solution of 20% DMSO was used to dissolve the compounds and was used as a negative control. Oxacillin and chloramphenicol were used as positive controls for *S. aureus*, while gentamicin and chloramphenicol were used as positive controls for *E. coli*. Both gentamicin and chloramphenicol were tested at 50 µg/disks, while oxacillin was used in 5 µg/disks. Each assay was conducted in duplicates and the antibacterial activities were measured as the diameter of the inhibition zone (mm ± SD). The disk diameter was 6 mm.

#### 3.9.4. α-Glucosidase Inhibitor Assay

α-Glucosidase enzyme was provided by the Tropical Biopharmaca Research Center, IPB University. α-Glucosidase inhibitor assay [40] was performed in a 96-well plate containing the reaction mixture of 50 μL of 0.1 M phosphate buffer (pH 7.0), 25 μL of 10 mM 4-nitrophenyl α-D-glucopyranoside (PNPG) which was dissolved in 0.1 M of phosphate buffer (pH 7.0), 10 μL of the test samples (each sample was diluted by DMSO), and 25 μL of α-glucosidase solutions (a stock solution of 1 mg/mL in 0.01 M of phosphate buffer with a pH of 7.0 was diluted to 0.04 U/mL with the same buffer just before assay). The reaction mixture was then incubated at 37 °C for 30 min. The reaction was terminated by the addition of 100 µL of 0.2 M of Na_2_CO_3_ solution. The enzymatic hydrolysis of substrate was monitored by the amount of PNPG released in the reaction mixture at 410 nm using a microplate reader. All the experiments were carried out in triplicates. Acarbose was used as a positive control, while DMSO as a negative control. The result was analyzed using MS Excel to obtain the IC_50_ value with its SD.

### 3.10. Computational Elucidation of the Molecular Mechanism of Manzamines

#### 3.10.1. Protein Target Identification

The direct protein targets of **1**–**3** were predicted using the PASS Targets web server available through the Way2Drug platform (https://way2drug.com/passtargets/) (3 October 2025). PASS Targets is a ligand-based computational system designed for predicting interactions between drug-like molecules and human proteins based on machine learning models generated from experimentally annotated bioactivity datasets derived from ChEMBL. The prediction framework employs a Naïve Bayes classification approach integrated with structure–activity relationship (SAR) information for identifying potential compound–protein interactions [41]. The platform evaluated compounds’ interaction probabilities against human protein targets and reported prediction confidence scores representing the difference between the probability of interaction and non-interaction for each target. We screened the results based on the confidence score.

#### 3.10.2. Protein-Protein Network and GO Enrichment Analysis

The target proteins from the previous analysis were submitted to the STRING database (https://string-db.org/) (2 January 2026) using the Multiple Protein menu. The analysis was configured for the organism Homo sapiens, with network edges set to “confidence” and a minimum required interaction score of 0.400 (medium confidence). After the network was updated based on these settings, the visualization results were exported as a tab-separated values (TSVs) file from the table/export menu.

#### 3.10.3. Molecular Docking

Three-dimensional structures of the target proteins were retrieved from the Protein Data Bank (PDBID: 7U31) in PDB format. Protein structures were selected based on structural resolution, completeness of amino acid residues, and the presence of experimentally resolved binding regions. The chemical structure of **1**–**3** were obtained from publicly available chemical databases in Structure Data File (SDF) format and imported into PyRx virtual screening software (PyRx version 0.8). Ligand preparation was conducted using the integrated Open Babel module available in PyRx. Energy minimization was performed using the MMFF94 force field to obtain the most stable molecular conformation before docking analysis. Molecular docking analysis was carried out using PyRx version 0.8, which integrates the AutoDock Vina (version 1.1.2) docking engine for protein–ligand interaction prediction. AutoDock Vina employs an empirical scoring function combined with gradient-based conformational search algorithms for estimating ligand binding affinity and generating optimal binding poses. The primary output of the docking simulation is the binding affinity (kcal/mol), representing the energy of the interaction between the ligand and protein. The resulting molecular interactions were visualized using BIOVIA Discovery Studio (version 25.1.0).

## 4. Conclusions

In conclusion, this study shows that Indonesian specimens of *Acanthostrongylophora ingens*, collected from four different localities, are a rich source of unique manzamine alkaloids. LC-HR-ESI-MS/MS and molecular networking revealed both known molecules and likely biosynthetic intermediates that support a unified manzamine biosynthetic pathway presented in this study. It also demonstrates that NMR calculations, together with X-ray crystallography, provide a powerful platform to resolve the stereochemistry of flexible marine alkaloids, while biological and computational assays identify manzamine A hydrochloride (**1**) as the most active isolate and GSK-3β as a plausible anticancer target of the manzamines. Overall, the study further strengthens the case for Indonesian marine sponges as valuable bioresources for natural product discovery and for manzamines as promising leads for further biosynthetic studies and pharmacological and target-based drug development.

## Figures and Tables

**Table 1:** Evaluation of cytotoxic activities **1**–**3** against brine shrimp *A. salina* and human immortal kidney HEK293T cells.

Compounds	*A. salina* LC_50_ ± SD (µM)	HEK293T IC_50_ ± SD (µM)
(+)-**1**	0.041 ± 0.012	0.599 ± 0.057
(+)-**2**	3.589 ± 0.924	11.395 ± 1.758
(+)-**3**	1.331 ± 0.341	11.380 ± 0.600
swinholide A	0.102 ± 0.042	0.152 ± 0.047
latrunculin A	0.172 ± 0.057	N.T.
laulimalide	1.793 ± 0.300	0.100 ± 0.007
paclitaxel	0.089 ± 0.015	0.059 ± 0.006
doxorubicin	0.990 ± 0.042	0.106 ± 0.011

N.T. is not tested.

**Table 2:** Molecular docking results for **1**–**3** binding with GSK-3β.

Ligand	LC_50_ ± SD (µM)	IC_50_ ± SD (µM)	Binding Affinity (kcal/mol)
(+)-**1**	0.041 ± 0.012	0.599 ± 0.057	−9.8
(+)-**2**	3.589 ± 0.924	11.395 ± 1.758	−10.4
(+)-**3**	1.331 ± 0.341	11.380 ± 0.600	−10.6
native ligand	-	-	−7.6

## Data Availability

The original contributions presented in this study are included in the article/Supplementary Material. Further inquiries can be directed to the corresponding authors.

## Supplementary Materials

The following supporting information can be downloaded at https://www.mdpi.com/article/10.3390/md24090313/s1. Figure S1. ^1^H and ^13^C NMR as well as HR-ESI-TOF-MS spectra of 1 together with its TLC profile; Figure S2. ^1^H and ^13^C NMR as well as HR-ESI-TOF-MS spectra of **2** together with its TLC profile; Figure S3. 1H and 13C NMR of **3** together with its TLC profile; Figure S4. HR-ESI-MS/MS spectrum of (Z)-8-pyridin-3-yl) oct-5-en-1-aminium (**o**); Figure S5. HR-ESI-MS/MS spectrum of 3-dodecylpyridine (**p**); Figure S6. HR-ESI-MS/MS spectrum of bis (3-alkylpyridine) macrocycle (**h**); Figure S7. HR-ESI-MS/MS spectrum of halicylclamine (**i**); Figure S8. HR-ESI-MS/MS spectrum of **k**; Figure S9. HR-ESI-MS/MS spectrum of tryptamine (**m**); Figure S10. HR-ESI-MS/MS spectrum of manzamine B (**2**); Figure S11. HR-ESI-MS/MS spectrum of **n**; Figure S12. HR-ESI-MS/MS spectrum of manzamine A (**1**); Figure S13. HR-ESI-MS/MS spectrum of 32.33-dihydro-31-hydroxymanzamine A (**3**); Figure S14. Molecular networking generated based on HR-ESI-MS/MS data of four EtOAc extracts of *Acanthostrongylophora ingens*; Figure S15. Chromatogram of A. ingens EtOAc extract from Sangiang island both positive and negative modes; Figure S16. Chromatogram of A. ingens EtOAc extract from Spermonde island both positive and negative modes; Figure S17. Chromatogram of A. ingens EtOAc extract from Thousands Island both positive and negative modes; Figure S18. Chromatogram of A. ingens EtOAc extract from Raja Ampat in positive mode; Table S1. Results of LC-HR-ESI-MS/MS analysis from Sangiang; Table S2. Results of LC-HR-ESI-MS/MS analysis from Spermonde; Table S3. Results of LC-HR-ESI-MS/MS analysis from Thousand island; Table S4. Results of LC-HR-ESI-MS/MS analysis from Raja Ampat.
