# Supplementary material for: Proposed Biosynthesis and Molecular Targets of Manzamine Alkaloids from Acanthostrongylophora ingens (Phylum Porifera: Class Demospongiae)
Source: Mar Drugs. 2026 Sep 7;24(9):313. doi: 10.3390/md24090313 (PMC13608748; doi:10.3390/md24090313)
Supplement: Supplementary file 1 [file marinedrugs-24-00313-s001.zip › marinedrugs-4465809-supplementary.pdf]

SUPPLEMENTARY MATERIAL

Proposed Biosynthesis and Molecular Targets of Manzamine Alkaloids from *Acanthostrongylophora ingens*

Novriyandi Hanif [1,2,*], Ritha Lusian Karuwal [1–3], Muhammad Naufal Khotmi Ramadhan Ashari Jaya Sudrajat [1], Didik Huswo Utomo [4], Gibral Abdul Khalik [1], Heri Yanti [1], Lik Tong Tan [5], Nicole J. de Voogd [6,7], Anggia Murni [2], Junichi Tanaka [8]

[1] Department of Chemistry, Faculty of Mathematics and Natural Sciences, IPB University, Bogor 16680 Indonesia;
[2] Tropical Biopharmaca Research Center, IPB University, Bogor 16128. Indonesia;
[3] Biology Education Program, Faculty of Teaching and Education, University of Pattimura, Ambon 97233 Indonesia;
[4] Bioinformatics Research Centers, Indonesian Institute of Bioinformatics (INBIO Indonesia), Malang 65145. Indonesia;
[5] Natural Sciences and Science Education, National Institute of Education, Nanyang Technological University, 1 Nanyang Walk, Singapore 637616, Singapore;
[6] Institute of Environmental Sciences (CML) Leiden University, 2300 RA Leiden, The Netherlands;
[7] Naturalis Biodiversity Center, 2333BE Leiden, The Netherlands;
[8] Department of Chemistry, Biology and Marine Science, University of the Ryukyus, Okinawa 903-0213, Japan
*Correspondence: nhanif@apps.ipb.ac.id; Tel.: +62-251-862-4567

Table of Contents

Title	Page
**Figure S1.** ^{1}H and ^{13}C NMR as well as HR-ESI-TOF-MS spectra of **1** together with its TLC profile.	**3**
**Figure S2.** ^{1}H and ^{13}C NMR as well as HR-ESI-TOF-MS spectra of 2 together with its TLC profile.	**4**
**Figure S3**. ^{1}H and ^{13}C NMR of **3** together with its TLC profile.	**5**
**Figure S4**. HR-ESI-MS/MS spectrum of (Z)-8-pyridin-3-yl) oct-5-en-1-aminium (**o**).	**6**
**Figure S5.** HR-ESI-MS/MS spectrum of 3-dodecylpyridine (**p**).	**6**
**Figure S6.** HR-ESI-MS/MS spectrum of bis (3-alkylpyridine) macrocycle (**h**).	**7**
**Figure S7.** HR-ESI-MS/MS spectrum of halicylclamine (**i**).	**7**
**Figure S8.** HR-ESI-MS/MS spectrum of **k.**	**8**
**Figure S9.** HR-ESI-MS/MS spectrum of tryptamine (**m**).	**8**
**Figure S10.** HR-ESI-MS/MS spectrum of manzamine B (**2**).	**9**
**Figure S11.** HR-ESI-MS/MS spectrum of **n.**	**9**
**Figure S12.** HR-ESI-MS/MS spectrum of manzamine A (**1**).	**10**
**Figure S13.** HR-ESI-MS/MS spectrum of 32.33-dihydro-31-hydroxymanzamine A (**3**).	**10**
**Figure S14.** Molecular networking generated based on HR-ESI-MS/MS data of four EtOAc extracts of *Acanthostrongylophora ingens*.	**11**
**Figure S15.** Chromatogram of *A. ingens* EtOAc extract from Sangiang island both positive and negative modes.	**12**
**Figure S16.** Chromatogram of *A. ingens* EtOAc extract from Spermonde island both positive and negative modes.	**13**
**Figure S17.** Chromatogram of *A. ingens* EtOAc extract from Thousands Island both positive and negative modes.	**13**
**Figure S18**. Chromatogram of *A. ingens* EtOAc extract from Raja Ampat in positive mode.	**14**
**Table S1.** Results of LC-HR-ESI-MS/MS analysis from Sangiang.	**15**
**Table S2.** Results of LC-HR-ESI-MS/MS analysis from Spermonde.	**17**
**Table S3.** Results of LC-HR-ESI-MS/MS analysis from Thousand island.	**19**
**Table S4.** Results of LC-HR-ESI-MS/MS analysis from Raja Ampat.	**20**

a) ^{1}H NMR of **1** ($CDCl_3$, 500 MHz)

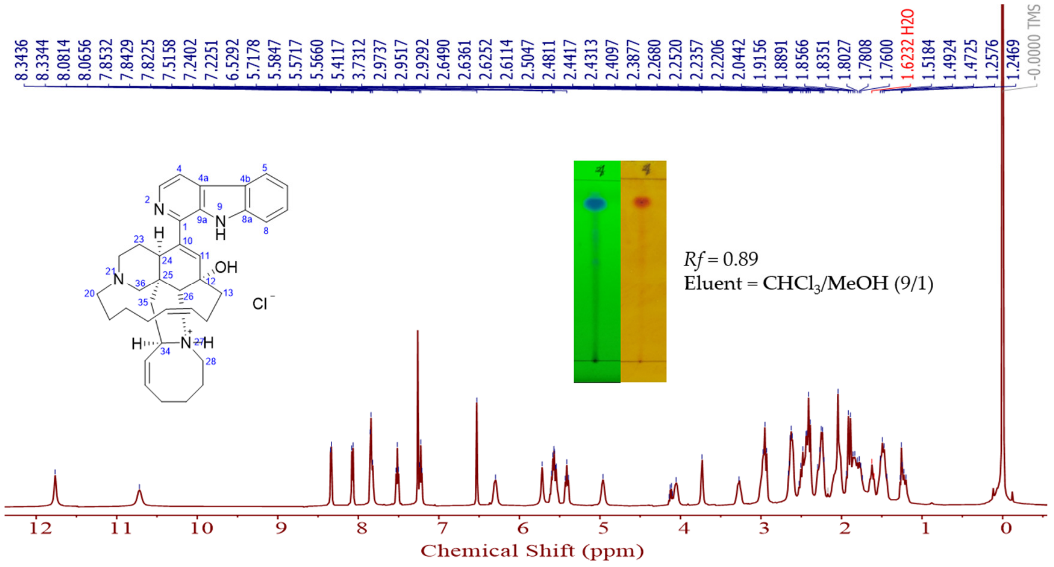

b) ^{13}C NMR of **1** ($CDCl_3$, 125 MHz)

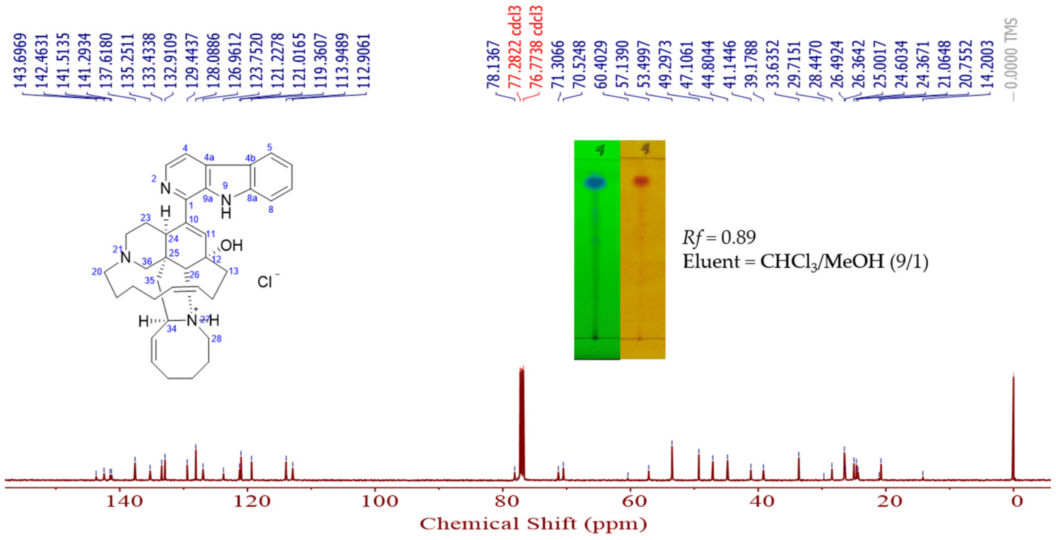

c) HR-ESI-TOF-MS (negative mode)

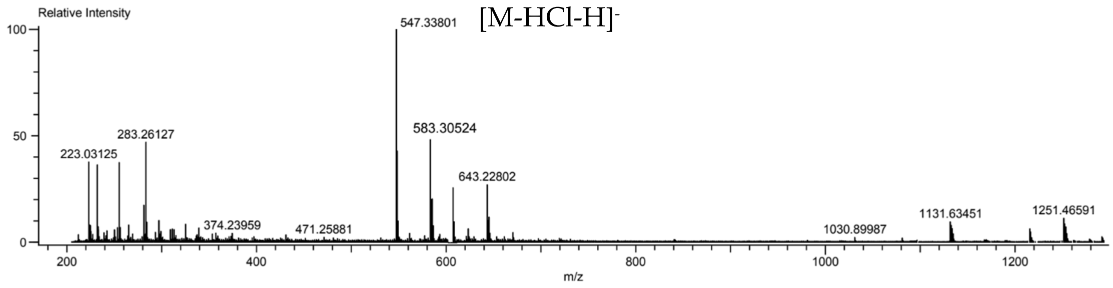

Figure S1. ^{1}H and ^{13}C NMR as well as HR-ESI-TOF-MS spectra of **1** together with its TLC profile.

a) ^{1}H NMR of **2** ($CDCl_3$, 500 MHz)

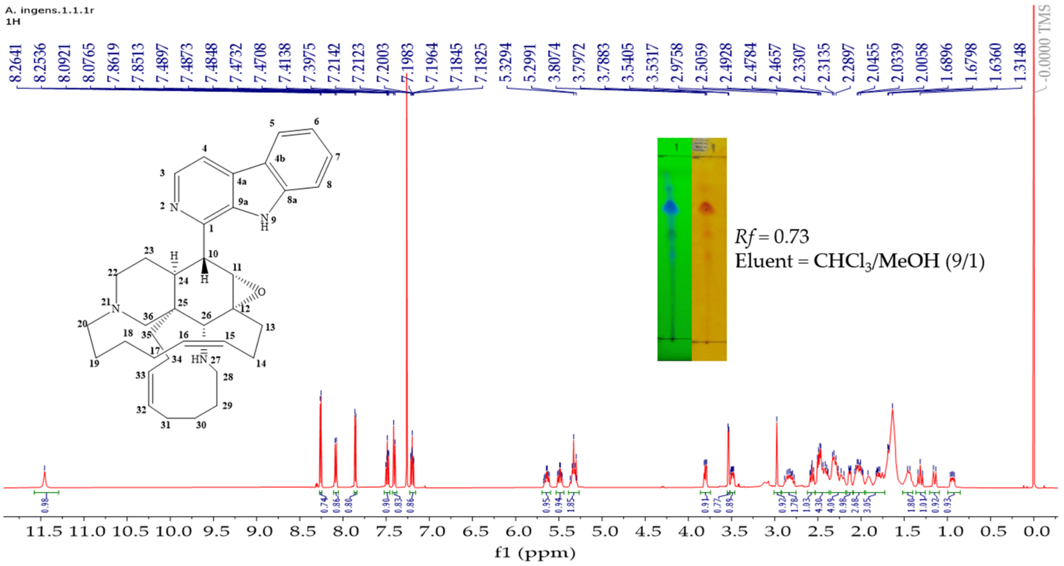

b) ^{13}C NMR of **2** ($CDCl_3$, 125 MHz)

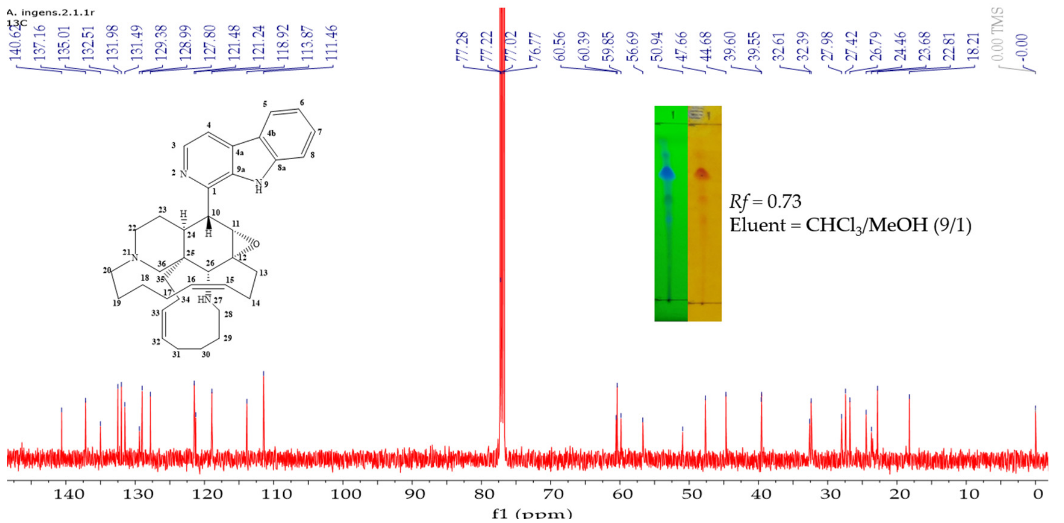

c) HR-ESI-TOF-MS (positive mode)

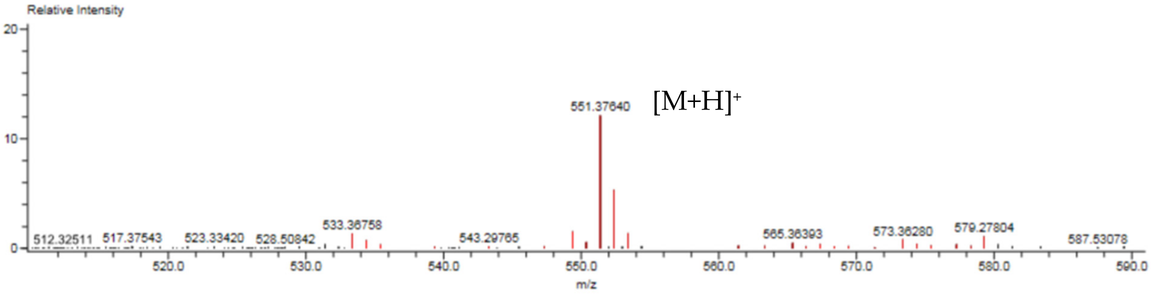

Figure S2. ^{1}H and ^{13}C NMR as well as HR-ESI-TOF-MS spectra of **2** together with its TLC profile.

a) ^{1}H NMR of **3** ($CDCl_3$, 500 MHz)

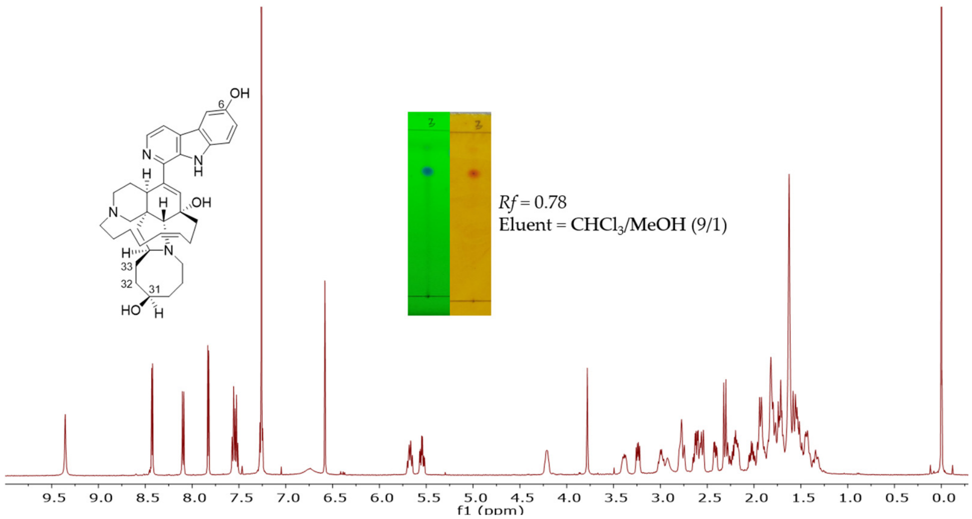

b) ^{13}C NMR of **3** ($CDCl_3$, 125 MHz)

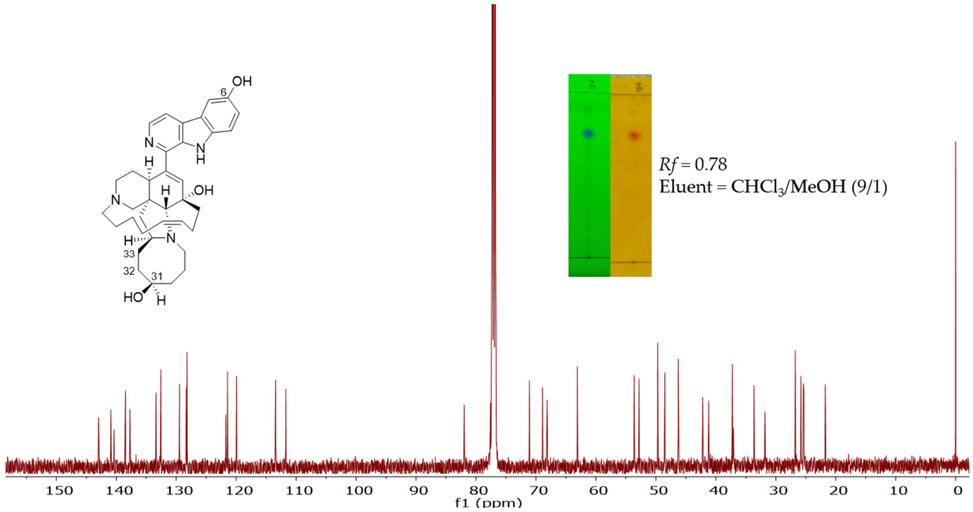

Figure S3. ^{1}H and ^{13}C NMR spectra of **3** together with its TLC profile.

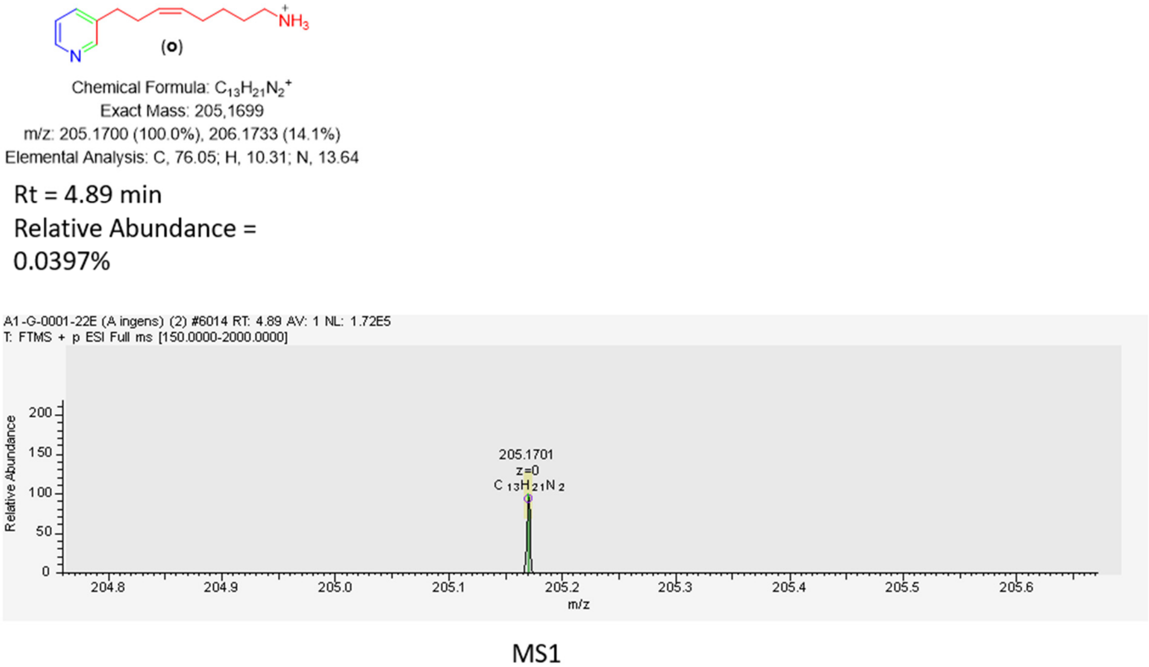

Figure S4. HR-ESI-MS/MS spectrum of (*Z*)-8-pyridin-3-yl) oct-5-en-1-aminium (**o**).

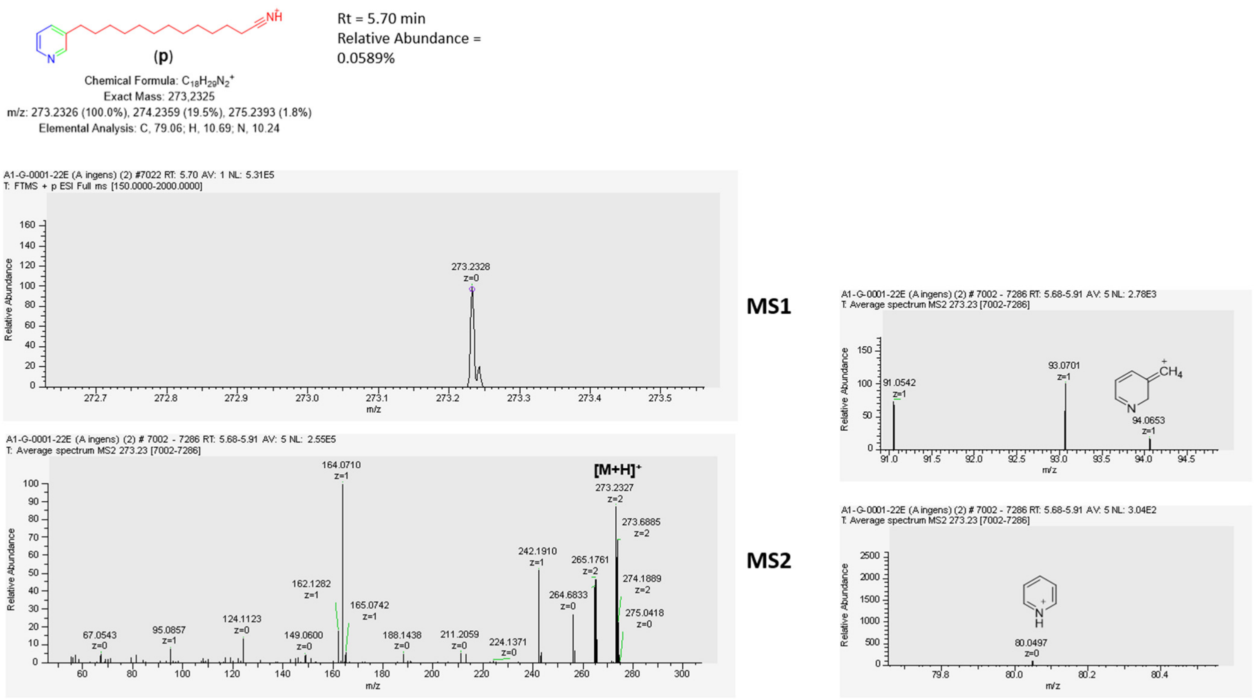

Figure S5. HR-ESI-MS/MS spectrum of 3-dodecylpyridine (**p**).

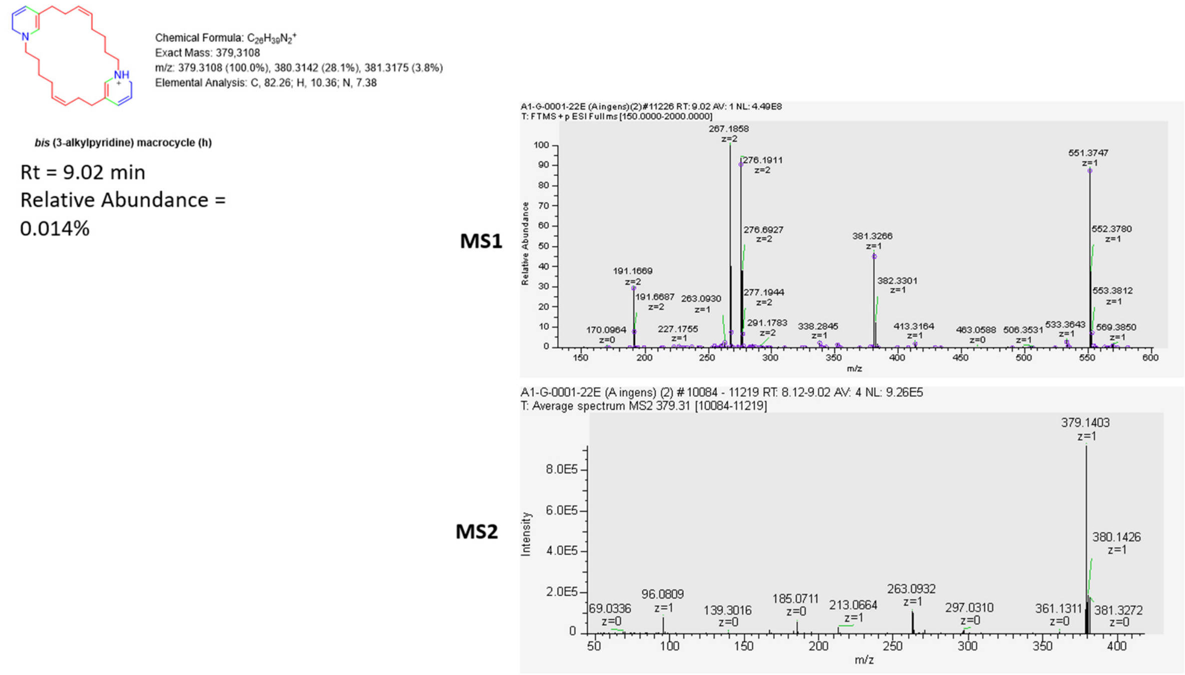

Figure S6. HR-ESI-MS/MS spectrum of *bis* (3-alkylpyridine) macrocycle (**h**).

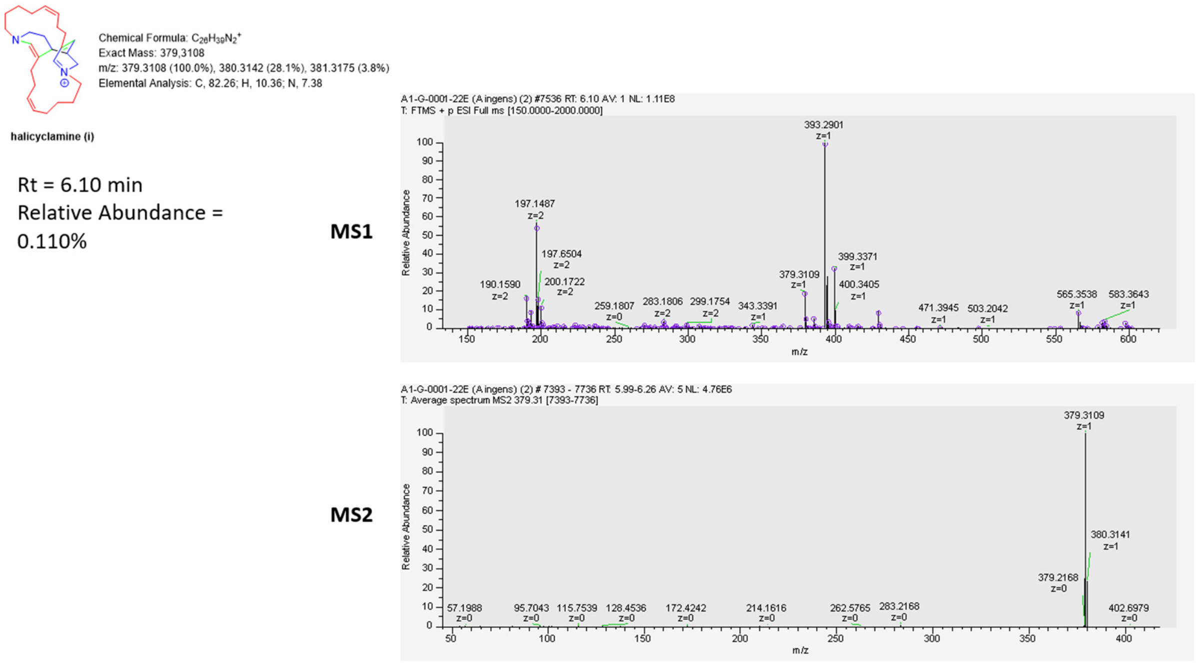

Figure S7. HR-ESI-MS/MS spectrum of halicylclamine (**i**).

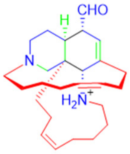

Chemical Formula: $C_{26}H_{41}N_2O^+$
Exact Mass: 397,3213
m/z: 397.3214 (100.0%), 398.3247 (28.1%), 399.3281 (3.8%)
Elemental Analysis: C, 78.54; H, 10.39; N, 7.05; O, 4.02

(**k**)

Rt = 4.75 min
Relative Abundance = 0.031%

MS1

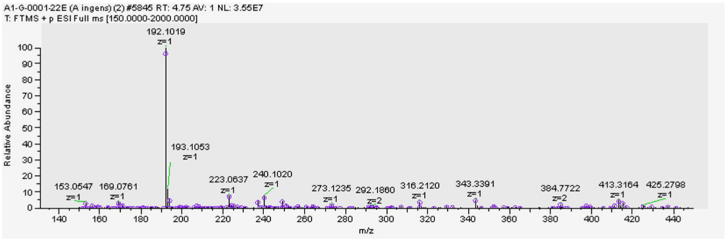

MS2

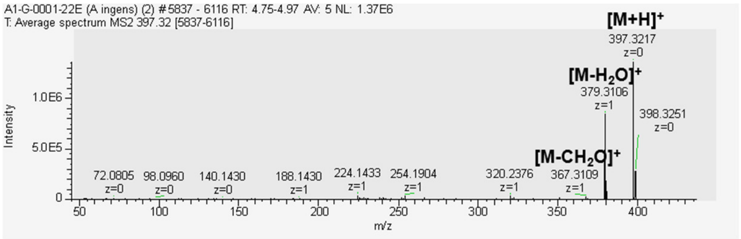

Figure S8. HR-ESI-MS/MS spectrum of **k**.

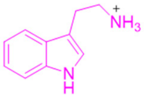

Chemical Formula: $C_{10}H_{13}N_2^+$
Exact Mass: 161,1073
m/z: 161.1074 (100.0%), 162.1107 (10.8%)
Elemental Analysis: C, 74.50; H, 8.13; N, 17.38

(**m**)

Rt = 5.16 min
Relative Abundance = 0.0040%

MS1

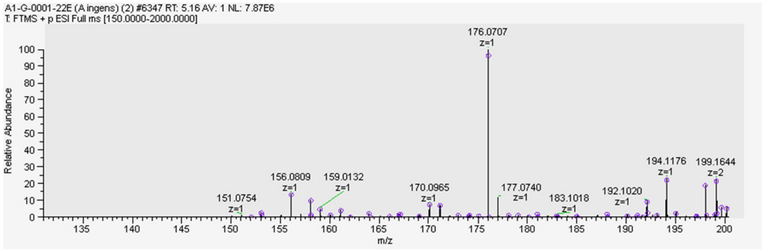

MS2

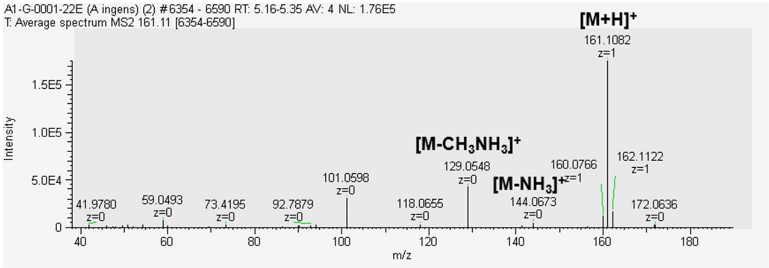

Figure S9. HR-ESI-MS/MS spectrum of tryptamine (**m**).

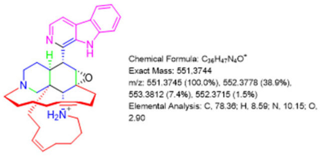

manzamine B (**2**)

Rt = 8.77 min
Relative Abundance = 1.63%

MS1

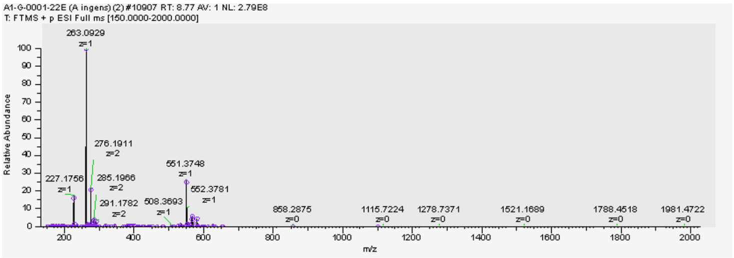

MS2

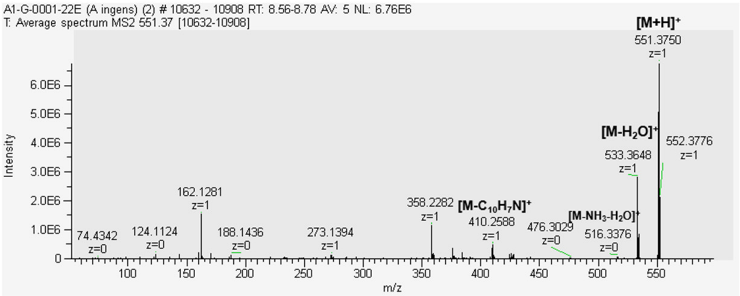

$C_{10}H_7N$ =

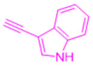

Figure S10. HR-ESI-MS/MS spectrum of manzamine B (**2**).

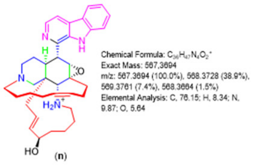

Rt = 9.2 min
Relative Abundance = 0.900%

MS1

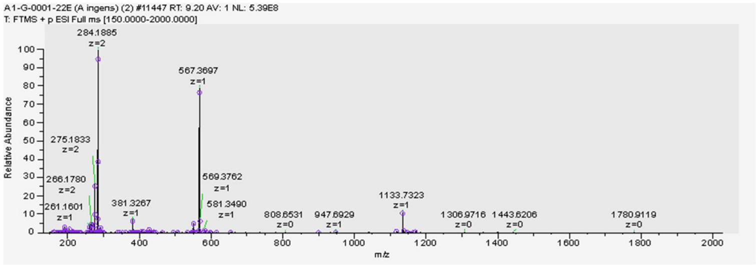

MS2

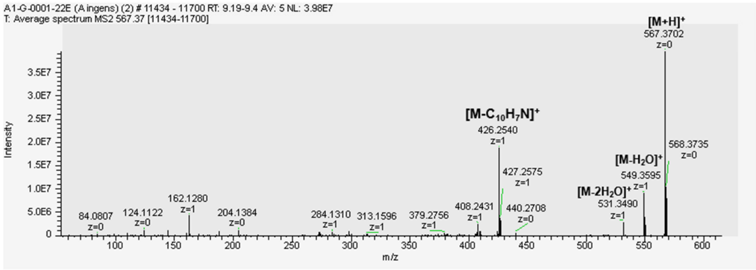

$C_{10}H_7N$ =

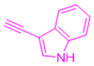

Figure S11. HR-ESI-MS/MS spectrum of **n**.

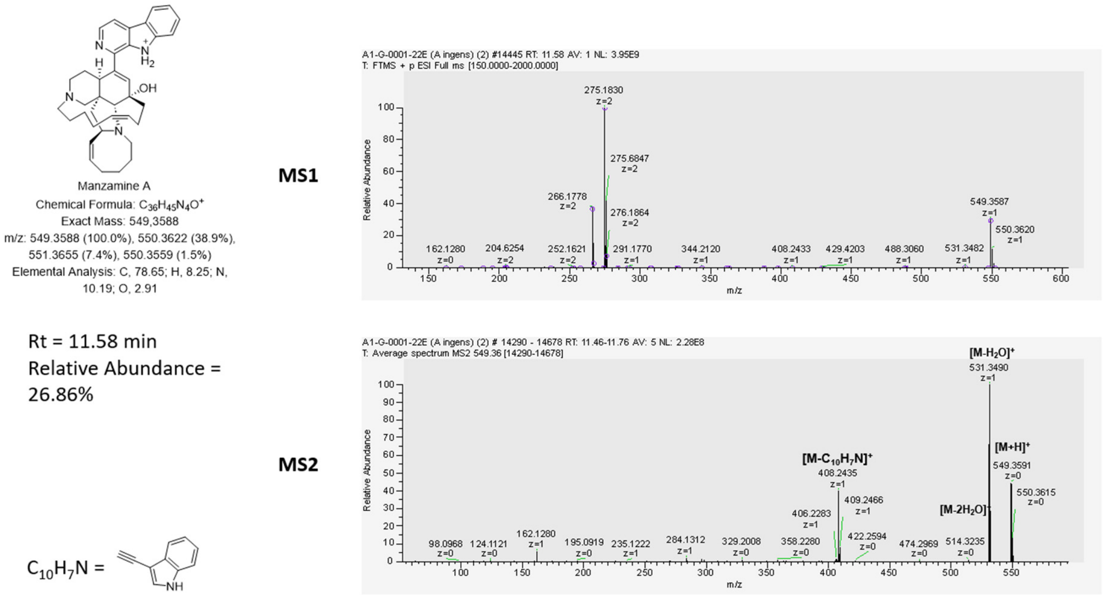

Figure S12. HR-ESI-MS/MS spectrum of manzamine A (**1**).

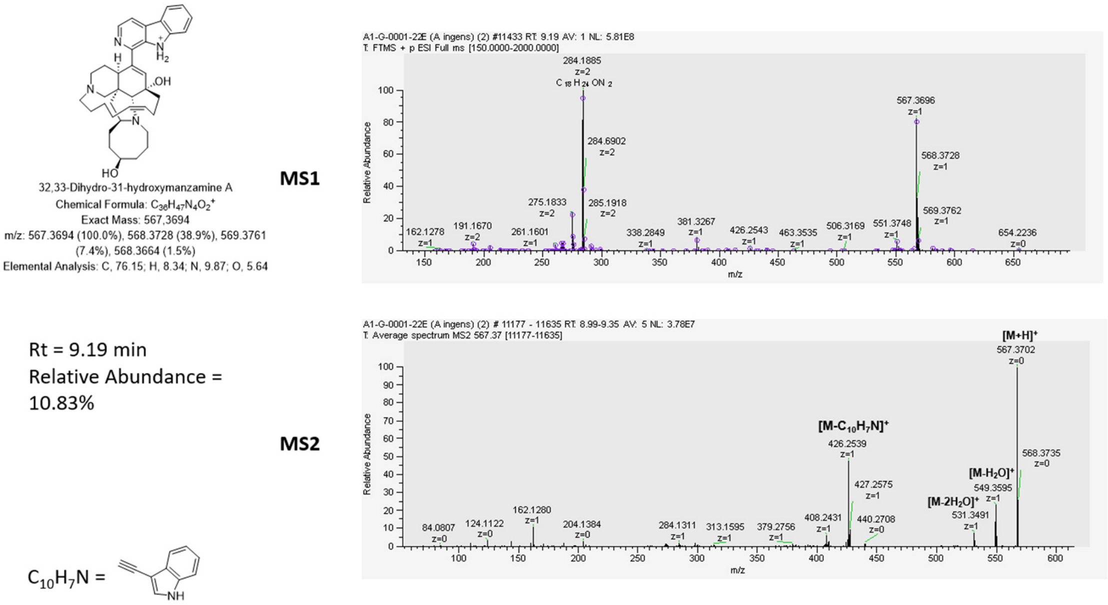

Figure S13. HR-ESI-MS/MS spectrum of 32.33-dihydro-31-hydroxymanzamine A (**3**).

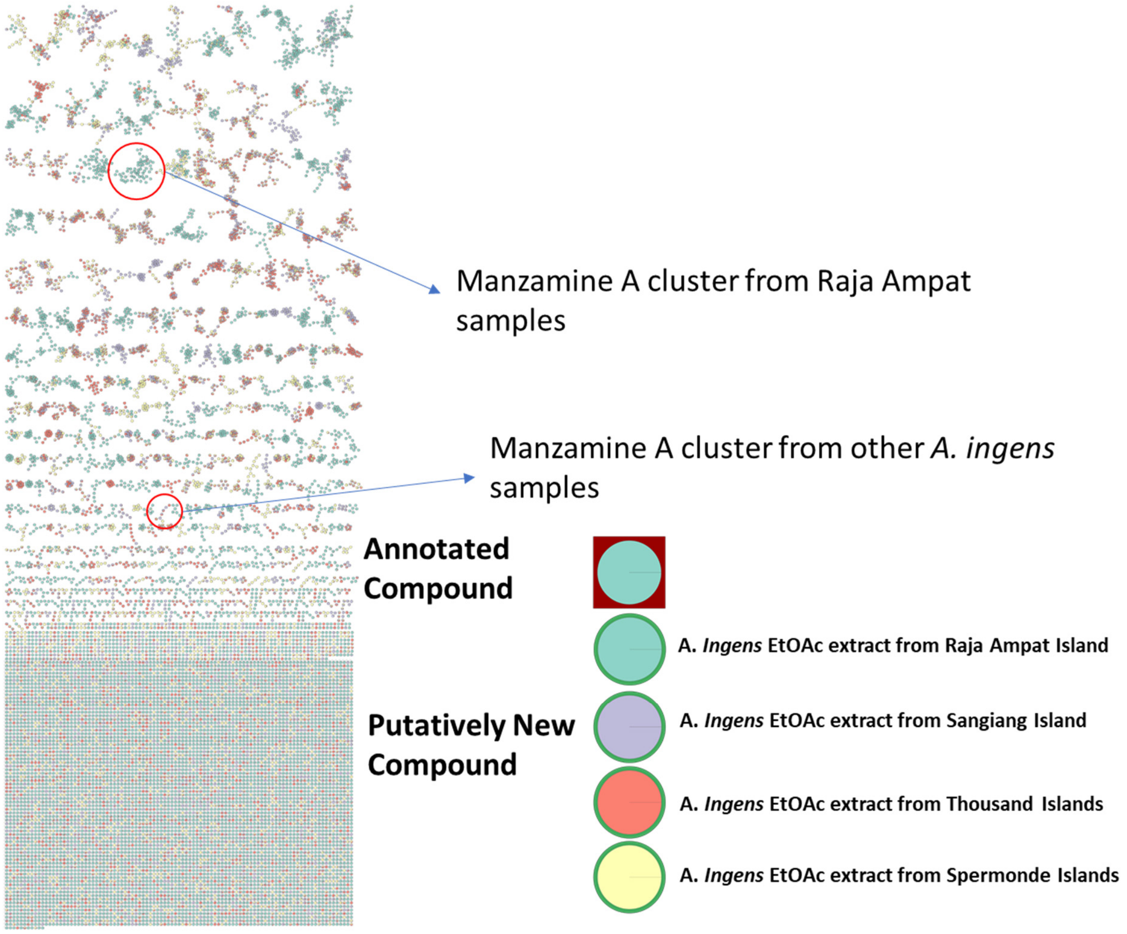

Figure S14. Molecular networking generated based on HR-ESI-MS/MS data of four EtOAc extracts of *Acanthostrongylophora ingens*.

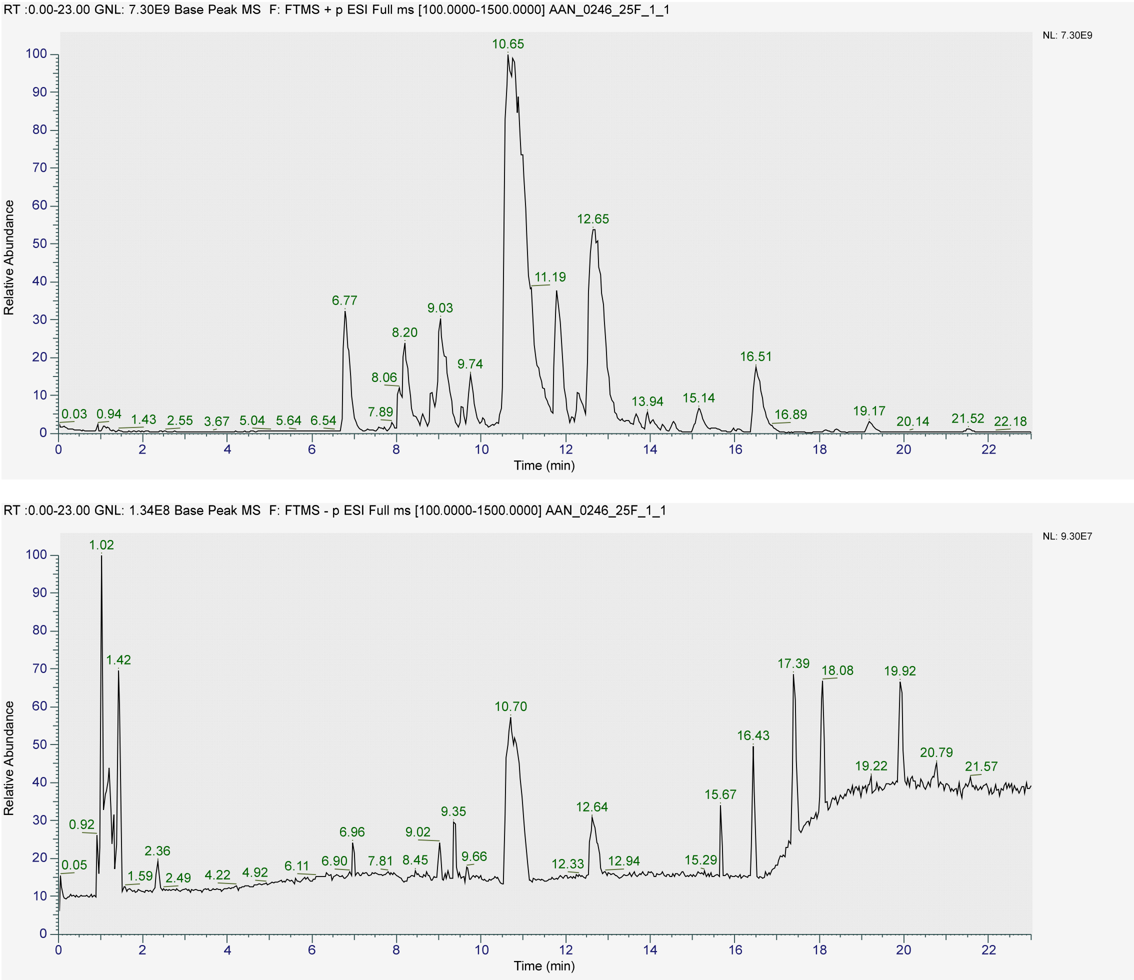

Figure S15. Chromatogram of *A. ingens* EtOAc extract from Sangiang island both positive and negative modes.

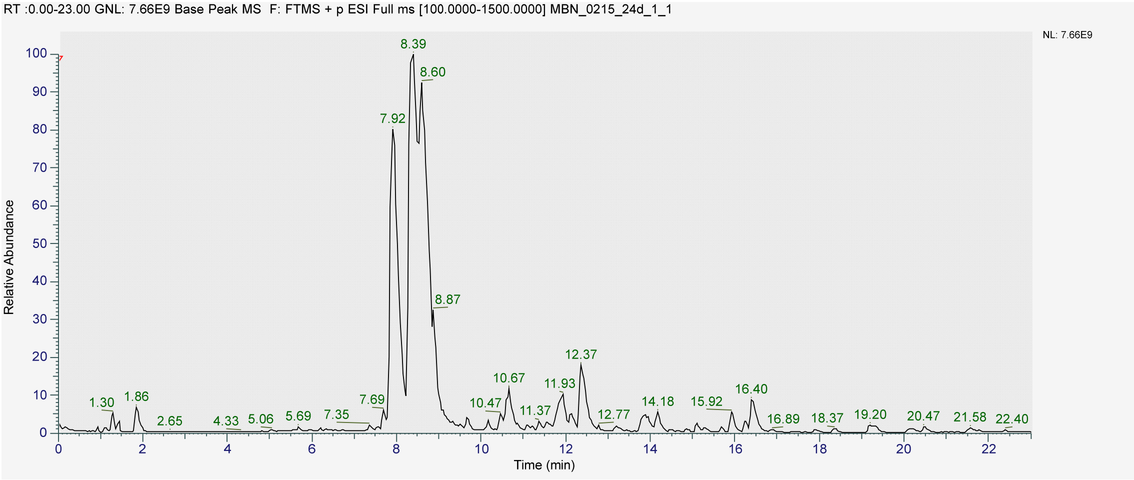

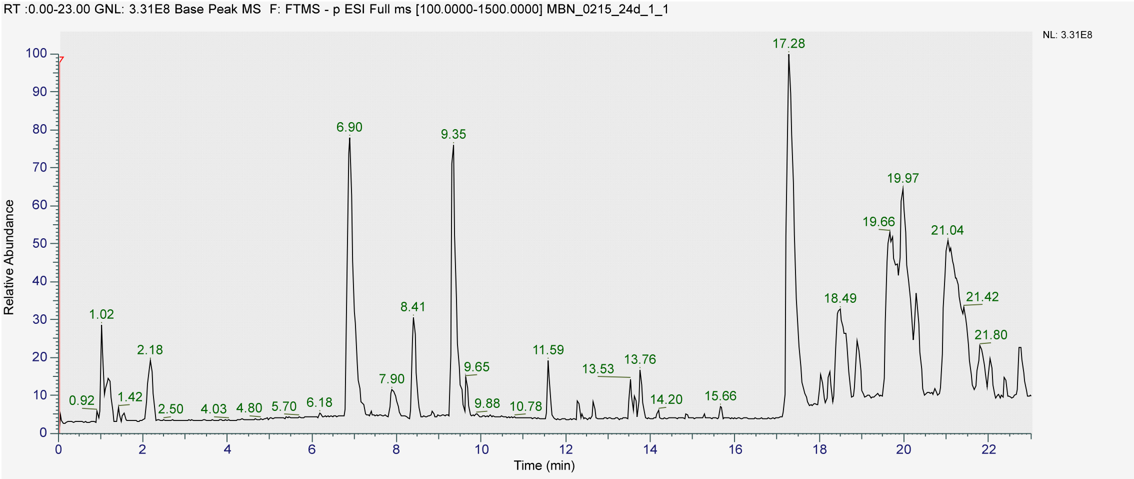

Figure S16. Chromatogram of *A. ingens* EtOAc extract from Spermonde island both positive and negative modes.

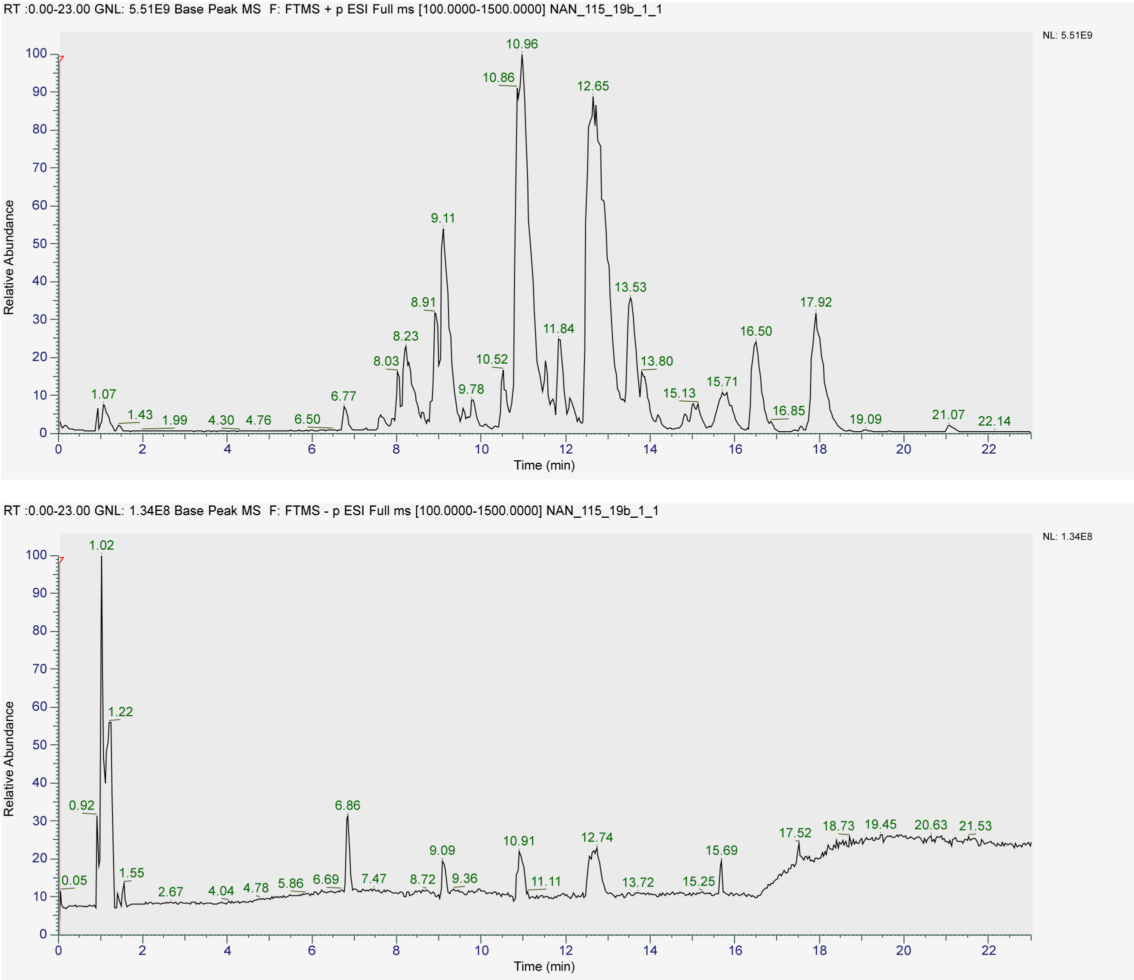

Figure S17. Chromatogram of *A. ingens* EtOAc extract from Thousands Island both positive and negative modes.

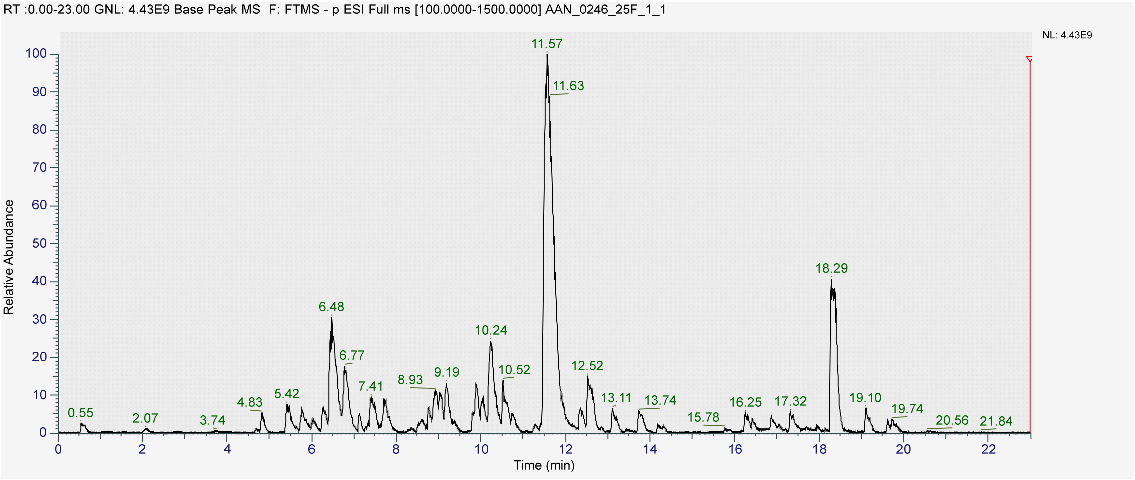

Figure S18. Chromatogram of *A. ingens* EtOAc extract from Raja Ampat in positive mode.

Table S1. Results of LC-HR-ESI-MS/MS analysis from Sangiang island.

No	Peak Mass	Formula Predicted	Theoretical Mass	Error (ppm)	RT	ESI Mode	Identification using MarinLit
1	304.2271	$C_{19}H_{30}O_2N$	304.2271	+0.02	13.94	+	NIL
2	303.2433	$C_{19}H_{31}ON_2$	303.2431	+0.65	14.56	+	NIL
3	395.3057	$C_{26}H_{39}ON_2$	395.3057	+0.09	15.14	+	zamamiphidin A (manzamine related)
4	332.2584	$C_{21}H_{34}O_2N$	332.2584	+0.05	15.96	+	NIL
5	302.3053	$C_{18}H_{40}O_2N$	302.3054	-0.29	17.44	+	NIL
6	304.2997	$C_{21}H_{38}N$	304.2999	-0.54	19.17	+	NIL
7	619.4029	$C_{29}H_{51}O_5N_{10}$	619.4038	-1.53	21.36	+	NIL
8	332.3310	$C_{23}H_{42}N$	332.3312	-0.64	21.52	+	NIL
9	531.3500	$C_{26}H_{49}O_8N_3$	531.3514	-2.71	21.52	+	NIL
10	463.3629	$C_{25}H_{51}O_7$	463.3629	-0.03	23.19	+	NIL
11	485.3447	$C_{23}H_{45}O_5N_6$	485.3446	+0.15	23.19	+	NIL
12	284.3310	$C_{19}H_{42}N$	284.3312	-0.54	23.50	+	NIL
13	398.2325	$C_{24}H_{32}O_4N$	398.2326	-0.18	24.50	+	NIL
14	457.2925	$C_{26}H_{39}O_4N_3$	457.2935	-2.11	24.50	+	NIL
15	515.3563	$C_{26}H_{49}O_7N_3$	515.3565	-0.47	24.50	+	NIL
16	256.2635	$C_{16}H_{34}ON$	256.2635	-0.01	24.88	+	NIL
17	433.2929	$C_{24}H_{39}O_4N_3$	433.2935	-1.46	24.88	+	NIL
18	497.2883	$C_{28}H_{39}O_5N_3$	497.2884	-0.31	24.88	+	NIL
19	311.1641	$C_{20}H_{23}O_3$	311.1642	-0.15	25.13	+	NIL
20	282.2790	$C_{18}H_{36}ON$	282.2791	-0.53	25.37	+	NIL
21	483.3452	$C_{29}H_{45}O_3N_3$	483.3455	-0.81	25.37	+	NIL
22	391.2817	$C_{22}H_{37}O_3N_3$	391.2829	-3.24	25.85	+	NIL
23	383.2766	$C_{20}H_{37}O_4N_3$	383.2779	-3.33	26.02	+	NIL
24	459.3085	$C_{24}H_{39}O_3N_6$	459.3078	+1.49	26.02	+	NIL
25	369.2975	$C_{20}H_{39}O_3N_3$	369.2986	-3.08	26.22	+	NIL
26	413.3240	$C_{22}H_{43}O_4N_3$	413.3248	-1.87	26.22	+	NIL
27	459.3086	$C_{24}H_{39}O_3N_6$	459.3078	+1.69	26.22	+	NIL
28	339.2505	$C_{18}H_{33}O_3N_3$	339.2516	-3.49	26.36	+	NIL
29	497.3241	$C_{29}H_{43}O_4N_3$	497.3248	-1.37	26.97	+	NIL
30	354.3008	$C_{21}H_{40}O_3N$	354.3003	+1.36	26.97	+	NIL
31	284.3315	$C_{19}H_{42}N$	284.3312	+1.07	26.97	+	NIL
32	393.2975	$C_{22}H_{39}O_3N_3$	393.2986	-2.74	27.58	+	NIL
33	284.2947	$C_{18}H_{38}ON$	284.2948	-0.40	27.58	+	NIL
34	284.2947	$C_{18}H_{38}ON$	284.2948	-0.29	28.08	+	NIL
35	447.3079	$C_{25}H_{41}O_4N_3$	447.3092	-2.83	28.08	+	NIL
36	391.2841	$C_{24}H_{39}O_4$	391.2843	-0.51	29.34	+	NIL
37	413.2659	$C_{24}H_{35}O_3N_3$	413.2673	-3.37	29.34	+	NIL
38	803.5439	$C_{48}H_{73}O_7N_3$	803.5443	-0.52	29.34	+	NIL
39	454.2930	$C_{26}H_{38}O_3N_4$	454.2938	-1.79	29.34	+	NIL

40	568.4267	$C_{40}H_{56}O_2$	568.4275	-1.38	29.57	+	anhydromicromonol/4'-hydroxy-dihydro-β.β-caroten-4-one/cucumariaxanthin A
41	393.2976	$C_{20}H_{37}O_2N_6$	393.2973	+0.83	29.57	+	NIL
42	427.3571	$C_{29}H_{47}O_2$	427.3571	+0.21	29.85	+	NIL
43	151.0255	$C_5H_3O_2N_4$	151.0251	+3.25	1.32	-	NIL
44	151.0255	$C_5H_3O_2N_4$	151.0251	+2.95	1.42	-	NIL
45	287.0889	$C_{12}H_{11}O_3N_6$	287.0887	+0.64	2.36	-	NIL
46	121.0287	$C_7H_5O_2$	121.0284	+2.32	6.96	-	NIL
47	613.3765	$C_{39}H_{51}O_5N$	613.3762	+0.57	9.02	-	NIL
48	567.3708	$C_{36}H_{47}O_2N_4$	567.3694	+2.64	9.02	-	NIL
49	144.0448	C_9H_6ON	144.0444	+3.05	9.35	-	NIL
50	595.3660	$C_{40}H_{45}N_5$	595.3670	-1.65	10.66	-	NIL
51	549.3602	$C_{36}H_{45}ON_4$	549.3588	+2.62	10.66	-	manzamine A
52	595.3660	$C_{39}H_{49}O_4N$	595.3656	+0.70	10.80	-	NIL
53	593.3505	$C_{39}H_{47}O_4N$	593.3500	+0.85	12.64	-	NIL
54	547.3447	$C_{36}H_{43}ON_4$	547.3431	+2.90	12.64	-	kepulauamine A
55	293.1763	$C_{18}H_{21}N_4$	293.1761	+0.67	15.67	-	NIL
56	420.2508	$C_{24}H_{36}O_6$	420.2506	+0.29	16.43	-	NIL
57	374.2451	$C_{23}H_{34}O_4$	374.2452	-0.20	16.43	-	pregna-5.20-diene-3.7.11-triol 3-acetate/sarcoehrenin C/plakinidone/sarcotin J/cladosporisteroid C/acalycigorgin D/dysidphenol C/penitalarin A/sclerosteroid D/plakortolide C/4.12-bis-n-butanoylalcyopterosin O/sarcoehrenolide Q/15.20-dimethoxypuupehenol
58	265.1482	$C_7H_{19}O_4N_7$	265.1493	-4.19	17.39	-	NIL
59	360.2218	$C_{12}H_{32}O_8N_4$	360.2215	+0.93	18.08	-	NIL
60	362.2375	$C_{13}H_{30}O_4N_8$	362.2385	-2.66	19.92	-	NIL
61	507.3542	$C_{27}H_{47}O_5N_4$	507.3541	+0.30	23.21	-	NIL

Table S2. Results of LC-HR-ESI-MS/MS analysis from Spermonde islands.

No	Peak Mass	Formula Predicted	Theoretical Mass	Error (ppm)	RT	ESI Mode	Identification using MarinLit
1	124.0395	$C_6H_6O_2N$	124.0393	+1.67	1.30	+	NIL
2	124.0396	$C_6H_6O_2N$	124.0393	+2.04	1.44	+	NIL
3	146.0602	C_9H_8ON	146.0600	+0.75	5.06	+	NIL
4	349.1764	$C_{17}H_{19}N_9$	349.1758	+1.63	9.57	+	NIL
5	227.1756	$C_{12}H_{23}O_2N_2$	227.1754	+0.92	10.17	+	NIL
6	409.2858	$C_{26}H_{37}O_2N_2$	409.2850	+2.13	11.07	+	NIL
7	187.0867	$C_{11}H_{11}ON_2$	187.0866	+0.67	11.20	+	NIL
8	262.1089	$C_{15}H_{12}N_5$	262.1087	+0.75	11.37	+	NIL
9	413.3164	$C_{24}H_{39}ON_5$	413.3149	+3.61	11.93	+	NIL
10	436.2965	$C_{27}H_{38}O_2N_3$	436.2959	+1.43	12.14	+	NIL
11	440.3273	$C_{27}H_{42}O_2N_3$	440.3272	+0.26	12.77	+	NIL
12	397.3214	$C_{26}H_{41}ON_2$	397.3213	+0.02	13.21	+	Zamaphidin B/C
13	443.2179	$C_{25}H_{27}O_2N_6$	443.2190	-2.49	13.45	+	NIL
14	362.2694	$C_{22}H_{36}O_3N$	362.2690	+1.13	13.45	+	NIL
15	413.3167	$C_{26}H_{41}O_2N_2$	413.3163	+1.10	13.61	+	NIL
16	317.2593	$C_{20}H_{33}ON_2$	317.2587	+1.69	13.61	+	NIL
17	361.3577	$C_{24}H_{45}N_2$	361.3577	-0.08	13.88	+	NIL
18	429.4202	$C_{29}H_{53}N_2$	429.4203	-0.37	13.88	+	NIL
19	304.2271	$C_{19}H_{30}O_2N$	304.2271	-0.08	13.95	+	NIL
20	361.3576	$C_{24}H_{45}N_2$	361.3577	-0.33	13.95	+	NIL
21	290.2478	$C_{19}H_{32}ON$	290.2478	-0.24	14.18	+	NIL
22	314.2481	$C_{21}H_{32}ON$	314.2478	+0.75	14.45	+	NIL
23	331.2746	$C_{21}H_{35}ON_2$	331.2744	+0.53	14.66	+	NIL
24	421.1772	$C_{25}H_{21}ON_6$	421.1771	+0.12	14.86	+	NIL
25	274.2741	$C_{16}H_{36}O_2N$	274.2741	+0.08	15.10	+	NIL
26	318.3002	$C_{18}H_{40}O_3N$	318.3003	-0.31	15.27	+	NIL
27	395.3056	$C_{26}H_{39}ON_2$	395.3057	-0.14	15.27	+	zamamiphidin A
28	304.2634	$C_{20}H_{34}ON$	304.2635	-0.41	15.30	+	NIL
29	318.3001	$C_{18}H_{40}O_3N$	318.3003	-0.60	15.30	+	NIL
30	320.2585	$C_{20}H_{34}O_2N$	320.2584	+0.24	15.68	+	NIL
31	332.2583	$C_{21}H_{34}O_2N$	332.2584	-0.41	15.92	+	NIL
32	306.2791	$C_{20}H_{36}ON$	306.2791	-0.29	16.23	+	NIL
33	318.2789	$C_{21}H_{36}ON$	318.2791	-0.76	16.40	+	NIL
34	359.3057	$C_{23}H_{39}ON_2$	359.3057	+0.01	16.89	+	NIL
35	373.3215	$C_{24}H_{41}ON_2$	373.3213	+0.43	17.09	+	stellettamide B
36	302.3053	$C_{18}H_{40}O_2N$	302.3054	-0.09	17.47	+	NIL
37	354.2429	$C_{23}H_{32}O_2N$	354.2428	+0.29	17.68	+	NIL
38	332.2584	$C_{21}H_{34}O_2N$	332.2584	-0.04	17.92	+	NIL
39	270.3155	$C_{18}H_{40}N$	270.3155	-0.25	18.37	+	NIL
40	346.2738	$C_{22}H_{36}O_2N$	346.2741	-0.64	19.20	+	NIL

41	304.2998	$C_{21}H_{38}N$	304.2999	-0.34	19.30	+	NIL
42	346.2737	$C_{22}H_{36}O_2N$	346.2741	-0.90	19.30	+	NIL
43	286.3103	$C_{18}H_{40}ON$	286.3104	-0.38	20.20	+	NIL
44	332.3308	$C_{23}H_{42}N$	332.3312	-1.19	21.58	+	NIL
45	449.3470	$C_{24}H_{49}O_7$	449.3473	-0.52	21.78	+	NIL
46	449.3473	$C_{24}H_{49}O_7$	449.3473	+0.09	22.05	+	NIL
47	449.3470	$C_{24}H_{49}O_7$	449.3473	-0.52	22.40	+	NIL
48	305.2086	$C_{15}H_{25}ON_6$	305.2084	+0.50	22.95	+	NIL
49	463.3628	$C_{25}H_{51}O_7$	463.3629	-0.36	23.22	+	NIL
50	485.3444	$C_{22}H_{39}N_{13}$	485.3446	-0.41	23.22	+	NIL
51	360.3623	$C_{25}H_{46}N$	360.3625	-0.56	23.83	+	NIL
52	319.2243	$C_{18}H_{29}O_2N_3$	319.2254	-3.43	24.06	+	NIL
53	297.2424	$C_{18}H_{33}O_3$	297.2424	+0.03	24.06	+	NIL
54	398.2327	$C_{24}H_{32}O_4N$	398.2326	+0.20	24.51	+	NIL
55	457.2930	$C_{26}H_{39}O_4N_3$	457.2935	-1.18	24.51	+	NIL
56	256.2634	$C_{16}H_{34}ON$	256.2635	-0.25	24.88	+	NIL
57	433.2927	$C_{22}H_{37}O_3N_6$	433.2922	+1.15	24.88	+	NIL
58	333.2399	$C_{19}H_{31}O_2N_3$	333.2411	-3.63	25.01	+	NIL
59	393.2765	$C_{23}H_{33}N_6$	393.2761	0.86	25.01	+	NIL
60	311.1642	$C_{20}H_{23}O_3$	311.1642	-0.05	25.15	+	NIL
61	282.2789	$C_{18}H_{36}ON$	282.2791	-0.96	25.38	+	NIL
62	282.2791	$C_{18}H_{36}ON$	282.2791	-0.31	25.78	+	NIL
63	347.2557	$C_{20}H_{33}O_2N_3$	347.2567	-3.03	26.18	+	NIL
64	282.2216	$C_{20}H_{28}N$	282.2216	-0.14	26.28	+	dispyridine
65	497.3600	$C_{28}H_{45}O_2N_6$	497.3599	+0.39	26.41	+	NIL
66	497.3242	$C_{27}H_{41}O_3N_6$	497.3235	+1.51	26.71	+	NIL
67	497.3240	$C_{29}H_{43}O_4N_3$	497.3248	-1.55	26.98	+	NIL
68	467.3501	$C_{29}H_{45}O_2N_3$	467.3506	-1.14	26.98	+	NIL
69	406.2529	$C_{30}H_{32}N$	406.2529	+0.01	27.14	+	NIL
70	411.3259	$C_{28}H_{43}O_2$	411.3258	+0.41	27.47	+	NIL
71	284.2947	$C_{18}H_{38}ON$	284.2948	-0.18	27.61	+	NIL
72	399.3258	$C_{25}H_{41}ON_3$	399.3244	+3.56	27.61	+	NIL
73	310.3103	$C_{20}H_{40}ON$	310.3104	-0.45	27.77	+	NIL
74	409.3464	$C_{27}H_{43}N_3$	409.3452	+2.99	27.87	+	NIL
75	447.3078	$C_{25}H_{41}O_4N_3$	447.3092	-3.04	28.04	+	NIL
76	310.3102	$C_{20}H_{40}ON$	310.3104	-0.94	28.37	+	NIL
77	1412.9113	$C_{68}H_{114}O_{10}N_{23}$	1412.9114	-0.07	28.37	+	NIL
78	413.3412	$C_{26}H_{43}ON_3$	413.3401	+2.71	28.60	+	NIL
79	461.3235	$C_{26}H_{43}O_4N_3$	461.3248	-2.73	28.90	+	NIL
80	425.3412	$C_{29}H_{45}O_2$	425.3414	-0.59	29.00	+	NIL
81	461.3241	$C_{26}H_{43}O_4N_3$	461.3248	-1.54	29.00	+	NIL
82	517.3424	$C_{33}H_{45}O_3N_2$	517.3425	-0.12	29.00	+	NIL
83	391.2841	$C_{22}H_{37}O_3N_3$	391.2829	+3.00	29.36	+	NIL

84	803.5435	$C_{46}H_{71}O_6N_6$	803.5430	+0.62	29.36	+	NIL
85	427.3569	$C_{29}H_{47}O_2$	427.3571	-0.43	29.83	+	NIL
86	121.0287	$C_7H_5O_2$	121.0284	+2.26	6.90	-	NIL
87	469.3075	$C_{29}H_{37}N_6$	469.3074	+0.22	7.90	-	NIL
88	174.0555	$C_{10}H_8O_2N$	174.0550	+3.20	8.41	-	NIL
89	144.0448	C_9H_6ON	144.0444	+2.52	9.35	-	NIL
90	187.0972	$C_9H_{15}O_4$	187.0965	+3.97	9.65	-	NIL
91	227.0713	$C_{14}H_{11}O_3$	227.0703	+4.55	11.59	-	NIL
92	466.2850	$C_{17}H_{38}O_7N_8$	466.2858	-1.77	12.29	-	NIL
93	215.1288	$C_{12}H_{15}N_4$	215.1291	-1.50	12.39	-	NIL
94	466.2849	$C_{16}H_{32}O_2N_{15}$	466.2858	-1.82	12.39	-	NIL
95	227.0714	$C_{14}H_{11}O_3$	227.0703	+4.75	12.65	-	NIL
96	183.1022	$C_{10}H_{15}O_3$	183.1016	+3.62	13.53	-	NIL
97	295.1554	$C_{17}H_{19}ON_4$	295.1553	+0.09	13.63	-	NIL
98	229.1445	$C_{13}H_{17}N_4$	229.1448	-1.06	13.63	-	NIL
99	305.1187	$C_{23}H_{15}N$	305.1199	-3.89	14.20	-	NIL
100	243.1603	$C_{14}H_{19}N_4$	243.1604	-0.66	14.84	-	NIL
101	465.1685	$C_{28}H_{23}O_4N_3$	465.1683	+0.39	14.84	-	streptocarbazole B
102	311.1868	$C_{18}H_{23}ON_4$	311.1866	+0.61	15.28	-	NIL
103	293.1763	$C_{18}H_{21}N_4$	293.1761	+0.88	15.66	-	NIL
104	265.1481	$C_7H_{19}O_4N_7$	265.1493	-4.65	17.28	-	NIL
105	360.2218	$C_{13}H_{28}O_4N_8$	360.2228	-2.78	18.04	-	NIL
106	348.2217	$C_{12}H_{28}O_4N_8$	348.2228	-3.14	18.25	-	NIL
107	311.1689	$C_{11}H_{25}O_7N_3$	311.1687	+0.78	18.49	-	NIL
108	311.1690	$C_{11}H_{25}O_7N_3$	311.1687	+0.98	18.90	-	NIL
109	325.1846	$C_{12}H_{27}O_7N_3$	325.1844	+0.67	19.66	-	NIL
110	293.1795	$C_7H_{21}O_3N_{10}$	293.1793	+0.73	19.97	-	NIL
111	325.1845	$C_{12}H_{27}O_7N_3$	325.1844	+0.39	19.97	-	NIL
112	325.1846	$C_{12}H_{27}O_7N_3$	325.1844	+0.77	20.28	-	NIL
113	339.2002	$C_{13}H_{29}O_7N_3$	339.2000	+0.57	21.04	-	NIL
114	493.3387	$C_{26}H_{45}O_5N_4$	493.3385	+0.54	21.80	-	NIL
115	493.3388	$C_{26}H_{45}O_5N_4$	493.3385	+0.73	22.38	-	NIL
116	507.3541	$C_{27}H_{47}O_5N_4$	507.3541	+0.06	23.20	-	NIL
117	391.2624	$C_{25}H_{33}ON_3$	391.2618	+1.39	24.86	-	metacycloprodigiosin

Table S3. Results of LC-HR-ESI-MS/MS analysis from Thousand island.

No	Peak Mass	Formula Predicted	Theoretical Mass	Error (ppm)	RT	ESI Mode	Identification using MarinLit
1	151.0354	$C_3H_7O_5N_2$	151.03495	+3.20	0.94	+	NIL
2	155.0816	$C_7H_{11}O_2N_2$	155.0815	+0.64	1.07	+	norzooanemonin methyl ester
3	268.1042	$C_{10}H_{14}O_4N_5$	268.10403	+0.47	1.43	+	NIL
4	169.0609	$C_7H_9O_3N_2$	169.06077	+0.94	1.43	+	NIL
5	413.3168	$C_{26}H_{41}O_2N_2$	413.31626	+1.40	8.03	+	NIL
6	551.3754	$C_{36}H_{47}ON_4$	551.37444	+1.68	10.86	+	NIL
7	581.3499	$C_{36}H_{45}O_3N_4$	581.34862	+2.23	11.53	+	pre-neo-kauluamine
8	567.3707	$C_{38}H_{49}O_3N$	567.3707	-0.05	11.84	+	NIL
9	565.3917	$C_{37}H_{49}ON_4$	565.39009	+2.78	12.11	+	NIL
10	549.3599	$C_{36}H_{45}ON_4$	549.35879	+2.07	12.65	+	manzamine A
11	565.3550	$C_{36}H_{45}O_2N_4$	565.3537	+2.26	13.53	+	8-hydroxymanzamine A
12	563.3760	$C_{37}H_{47}ON_4$	563.37444	+2.84	13.80	+	NIL
13	551.3759	$C_{36}H_{47}ON_4$	551.37444	+2.57	14.17	+	NIL
14	549.3601	$C_{36}H_{45}ON_4$	549.35879	+2.40	14.82	+	manzamine A
15	581.3852	$C_{37}H_{49}O_2N_4$	581.385	+0.33	15.03	+	NIL
16	395.3059	$C_{26}H_{39}ON_2$	395.30569	+0.55	15.13	+	zamamiphidin A
17	567.4068	$C_{37}H_{51}ON_4$	567.40574	+1.87	16.50	+	NIL
18	318.2431	$C_{20}H_{32}O_2N$	318.24276	+1.00	16.85	+	NIL
19	334.2741	$C_{21}H_{36}O_2N$	334.27406	+0.16	17.57	+	NIL
20	302.3056	$C_{18}H_{40}O_2N$	302.30536	+0.62	17.40	+	NIL
21	533.3640	$C_{36}H_{45}N_4$	533.36387	+0.26	17.92	+	NIL
22	561.3585	$C_{37}H_{45}ON_4$	561.35879	-0.59	21.07	+	NIL
23	563.3370	$C_{34}H_{41}ON_7$	563.33671	+0.47	23.18	+	NIL
24	463.3622	$C_{25}H_{51}O_7$	463.36293	-1.48	23.18	+	NIL
25	398.2326	$C_{24}H_{32}O_4N$	398.23258	+0.05	24.53	+	NIL
26	411.3620	$C_{29}H_{47}O$	411.36214	-0.35	25.29	+	NIL
27	369.2974	$C_{20}H_{39}O_3N_3$	369.29859	-3.33	26.25	+	NIL
28	425.3629	$C_{26}H_{49}O_4$	425.36254	+0.89	26.25	+	NIL
29	394.3466	$C_{28}H_{44}N$	394.34683	-0.45	29.87	+	NIL
30	121.0287	$C_7H_5O_2$	121.02841	+2.44	6.86	-	NIL
31	595.3663	$C_{40}H_{45}N_5$	595.36695	-1.14	9.09	-	NIL
32	595.3663	$C_{39}H_{49}O_4N$	595.36561	+1.21	10.91	-	NIL
33	593.3505	$C_{39}H_{47}O_4N$	593.34996	+0.85	12.57	-	NIL
34	593.3505	$C_{37}H_{45}O_3N_4$	593.34862	+3.11	12.74	-	NIL
35	293.1763	$C_{18}H_{21}N_4$	293.17607	+0.77	15.69	-	NIL
36	265.1483	$C_7H_{19}O_4N_7$	265.1493	-3.84	17.52	-	NIL
37	507.3542	$C_{27}H_{47}O_5N_4$	507.3541	+0.24	23.19	-	amphiasterin C2/D2

Table S4. Results of LC-HR-ESI-MS/MS analysis from Raja Ampat.

No	Peak Mass	Formula Predicted	Theoretical Mass	Error (ppm)	RT	ESI Mode	Identification using MarinLit
1	202.1801	$C_{11}H_{24}O_2N$	202.1802	-0.05	0.55	+	NIL
2	265.0796	$C_{10}H_{11}O_4N_5$	265.0806	-3.64	1.06	+	NIL
3	243.0977	$C_{10}H_{15}O_5N_2$	243.0976	+0.45	1.06	+	NIL
4	427.2956	$C_{26}H_{39}O_3N_2$	427.2955	+0.24	1.56	+	NIL
5	302.1964	$C_{15}H_{28}O_5N$	302.1962	+0.60	1.56	+	NIL
6	227.0223	$C_{14}H_9O_2N$	223.0628	+4.35	1.56	+	NIL
7	244.1909	$C_{13}H_{26}O_3N$	244.1907	+0.75	1.56	+	NIL
8	249.1135	$C_{15}H_{13}N_4$	249.1135	+0.09	2.07	+	NIL
9	425.2799	$C_{26}H_{37}O_3N_2$	425.2799	+0.02	2.79	+	NIL
10	403.1513	$C_{21}H_{19}O_3N_6$	403.1513	-0.10	2.79	+	NIL
11	197.1284	$C_{10}H_{17}O_2N_2$	197.1285	+0.36	3.74	+	NIL
12	316.2119	$C_{16}H_{30}O_5N$	316.2119	+0.30	3.92	+	NIL
13	192.1019	$C_{11}H_{14}O_2N$	192.1019	-0.20	4.70	+	NIL
14	415.3318	$C_{26}H_{43}O_2N_2$	415.3319	-0.36	4.83	+	ircinol B
15	236.1828	$C_7H_{22}O_2N_7$	236.1830	-0.44	5.27	+	NIL
16	399.3369	$C_{26}H_{43}ON_2$	399.3370	-0.34	5.45	+	NIL
17	211.1441	$C_{11}H_{19}O_2N_2$	211.1441	-0.07	5.45	+	NIL
18	399.3370	$C_{26}H_{43}ON_2$	399.3370	+0.11	5.77	+	xestamine F
19	393.2901	$C_{26}H_{37}ON_2$	393.2900	+0.07	6.04	+	NIL
20	383.3422	$C_{26}H_{43}N_2$	383.3421	+0.23	6.28	+	NIL
21	553.3900	$C_{36}H_{49}ON_4$	553.3901	-0.24	7.14	+	NIL
22	567.3696	$C_{36}H_{47}O_2N_4$	567.3694	+0.38	7.27	+	32.33-dihydro-31-hydroxymanzamine A
23	581.3486	$C_{36}H_{45}O_3N_4$	581.3486	-0.29	7.41	+	pre-neo-kauluamine
24	583.3640	$C_{36}H_{47}O_3N_4$	583.3643	-0.54	7.51	+	32.33-dihydro-6.31-dihydroxymanzamine A
25	569.3850	$C_{34}H_{47}ON_7$	569.3837	+2.37	7.72	+	NIL
26	565.3542	$C_{36}H_{45}O_2N_4$	565.3537	+0.96	8.08	+	8-hydroxymanzamine A/31-hydroxymanzamine A/Manzamine E or its isomer
27	247.1443	$C_{14}H_{19}O_2N_2$	247.1441	+0.99	8.08	+	NIL
28	413.3167	$C_{26}H_{41}O_2N_2$	413.3163	+1.10	8.08	+	NIL
29	379.1404	$C_{20}H_{19}O_4N_4$	379.1401	+0.95	8.08	+	NIL
30	379.1405	$C_{20}H_{19}O_4N_4$	379.1401	+1.19	8.29	+	NIL
31	569.3855	$C_{36}H_{49}O_2N_4$	569.3850	+0.87	8.08	+	3.4-dihydromanzamine J N-oxide
32	379.1404	$C_{20}H_{19}O_4N_4$	379.1401	+0.95	8.36	+	NIL
33	551.3751	$C_{38}H_{49}O_2N$	551.3758	-1.31	8.29	+	NIL
34	551.3749	$C_{36}H_{47}ON_4$	551.3744	+0.80	8.36	+	NIL
35	555.4059	$C_{36}H_{51}ON_4$	555.4057	+0.26	8.53	+	NIL

36	567.3694	$C_{36}H_{47}O_2N_4$	567.3694	+0.16	8.53	+	manzamine J N-oxide/manzamine B N-oxide
37	567.3698	$C_{34}H_{45}ON_7$	567.3680	+3.07	8.62	+	NIL
38	263.0929	$C_{15}H_{11}ON_4$	263.0927	+0.72	8.77	+	NIL
39	563.3382	$C_{36}H_{43}O_2N_4$	563.3381	+0.25	8.85	+	12.28-oxamanzamine E
40	381.3264	$C_{26}H_{41}N_2$	381.3264	+0.29	8.93	+	neopetrocyclamine A
41	381.3266	$C_{26}H_{41}N_2$	381.3264	+0.53	9.02	+	neopetrocyclamine A
42	551.3747	$C_{36}H_{47}ON_4$	551.3744	+0.46	9.02	+	NIL
43	567.3696	$C_{36}H_{47}O_2N_4$	567.3694	+0.49	9.19	+	manzamine J N-oxide/manzamine B N-oxide
44	579.3333	$C_{36}H_{43}O_3N_4$	579.3330	+0.60	9.34	+	12.34-oxa-6-hydroxymanzamine E
45	595.3283	$C_{36}H_{43}O_4N_4$	595.3279	+0.72	9.34	+	acantholactam
46	595.3283	$C_{36}H_{43}O_4N_4$	595.3279	+0.72	9.63	+	acantholactone
47	565.3539	$C_{38}H_{47}O_3N$	565.3551	-1.95	9.82	+	NIL
48	551.3747	$C_{36}H_{47}ON_4$	551.3744	+0.46	9.89	+	NIL
49	565.3539	$C_{36}H_{45}O_2N_4$	565.3537	+0.32	10.06	+	8-hydroxymanzamine A/31-hydroxymanzamine A/Manzamine E or its isomer
50	429.4206	$C_{29}H_{53}N_2$	429.4203	+0.55	10.75	+	NIL
51	361.3580	$C_{24}H_{45}N_2$	361.3577	+0.68	10.75	+	NIL
52	565.3541	$C_{36}H_{45}O_2N_4$	565.3537	+0.75	10.24	+	8-hydroxymanzamine A/31-hydroxymanzamine A/Manzamine E or its isomer
53	565.3539	$C_{36}H_{45}O_2N_4$	565.3537	+0.75	10.75	+	8-hydroxymanzamine A/31-hydroxymanzamine A/Manzamine E or its isomer
54	569.3854	$C_{36}H_{49}O_2N_4$	569.3850	+0.65	11.29	+	NIL
55	549.3587	$C_{36}H_{45}ON_4$	549.3588	-0.15	11.57	+	manzamine A
56	565.3539	$C_{36}H_{45}O_2N_4$	565.3537	+0.32	12.36	+	8-hydroxymanzamine A/31-hydroxymanzamine A/Manzamine E or its isomer
57	415.2115	$C_{24}H_{31}O_6$	415.2115	-0.14	12.52	+	NIL
58	846.4423	$C_{48}H_{64}O_{12}N$	846.4423	+0.02	12.52	+	NIL
59	277.1799	$C_{17}H_{25}O_3$	277.1798	+0.40	13.11	+	NIL
60	259.1906	$C_{14}H_{27}O_4$	259.1904	+0.52	13.74	+	NIL
61	236.1647	$C_{14}H_{22}O_2N$	236.1645	+1.03	14.19	+	NIL
62	412.3789	$C_{25}H_{50}O_3N$	412.3785	+0.93	14.3	+	NIL
63	574.4736	$C_{38}H_{60}ON_3$	574.4731	+0.84	15.03	+	NIL
64	440.4102	$C_{27}H_{54}O_3N$	440.4098	+0.97	15.14	+	NIL
65	295.2245	$C_{16}H_{29}O_2N_3$	295.2254	-3.29	15.78	+	NIL
66	468.4411	$C_{29}H_{58}O_3N$	468.4411	+0.02	15.78	+	NIL
67	531.2744	$C_{33}H_{39}O_6$	531.2741	+0.45	15.88	+	NIL
68	313.2738	$C_{19}H_{37}O_3$	313.2737	+0.16	16.25	+	NIL
69	331.2845	$C_{19}H_{39}O_4$	331.2843	+0.60	16.25	+	NIL

70	463.3629	$C_{25}H_{51}O_7$	463.3629	+0.03	16.42	+	NIL
71	341.3048	$C_{21}H_{41}O_3$	341.3050	-0.53	16.87	+	NIL
72	359.3154	$C_{21}H_{43}O_4$	359.3156	-0.52	16.87	+	NIL
73	319.2632	$C_{21}H_{35}O_2$	319.2632	+0.18	16.95	+	NIL
74	338.3419	$C_{22}H_{44}ON$	338.3417	+0.52	17.32	+	NIL
75	338.3418	$C_{20}H_{42}N_4$	338.3404	+4.13	17.38	+	NIL
76	698.4991	$C_{41}H_{62}O_2N_8$	698.4990	+0.17	17.94	+	NIL
77	663.4534	$C_{44}H_{59}O_3N_2$	663.4520	+2.03	18.29	+	NIL
78	1342.9263	$C_{79}H_{122}O_{10}N_8$	1342.9278	-1.17	18.29	+	NIL
79	551.5035	$C_{35}H_{67}O_4$	551.5034	+0.28	19.1	+	NIL
80	586.5406	$C_{35}H_{72}O_5N$	586.5405	+0.15	19.1	+	NIL
81	647.4588	$C_{44}H_{59}O_2N_2$	647.4571	+2.62	19.63	+	NIL
82	579.5345	$C_{37}H_{71}O_4$	579.5347	-0.24	19.74	+	NIL
83	614.5716	$C_{35}H_{74}O_4N_4$	614.5705	+1.85	19.74	+	NIL
84	607.5663	$C_{39}H_{75}O_4$	607.5660	+0.49	20.56	+	NIL
85	642.6033	$C_{38}H_{74}N_8$	642.6031	+0.37	20.56	+	NIL
86	871.5730	$C_{54}H_{69}N_{11}$	871.5732	-0.64	21.82	+	NIL
87	871.5730	$C_{55}H_{75}O_5N_4$	871.5732	-0.65	21.82	+	NIL
88	1796.4850	$C_{114}H_{195}O_{11}N_4$	1796.4817	+1.83	21.82	+	NIL
